# Meta-proteomic analysis of protein expression distinctive to electricity-generating biofilm communities in air-cathode microbial fuel cells

**DOI:** 10.1186/s13068-018-1111-2

**Published:** 2018-04-23

**Authors:** Jeremy F. Chignell, Susan K. De Long, Kenneth F. Reardon

**Affiliations:** 1 Department of Chemical and Biological Engineering, Colorado State University, Fort Collins, USA; 2 Department of Civil and Environmental Engineering, Colorado State University, Fort Collins, USA; 3 Cell and Molecular Biology Graduate Program, Colorado State University, Fort Collins, USA

**Keywords:** Bioelectrochemical, Proteomics, Meta-proteomics, Microbial fuel cell, Metagenomics, Biofilm, Exoelectrogen, *Geobacter*

## Abstract

**Background:**

Bioelectrochemical systems (BESs) harness electrons from microbial respiration to generate power or chemical products from a variety of organic feedstocks, including lignocellulosic biomass, fermentation byproducts, and wastewater sludge. In some BESs, such as microbial fuel cells (MFCs), bacteria living in a biofilm use the anode as an electron acceptor for electrons harvested from organic materials such as lignocellulosic biomass or waste byproducts, generating energy that may be used by humans. Many BES applications use bacterial biofilm communities, but no studies have investigated protein expression by the anode biofilm community as a whole.

**Results:**

To discover functional protein expression during current generation that may be useful for MFC optimization, a label-free meta-proteomics approach was used to compare protein expression in acetate-fed anode biofilms before and after the onset of robust electricity generation. Meta-proteomic comparisons were integrated with 16S rRNA gene-based community analysis at four developmental stages. The community composition shifted from dominance by aerobic *Gammaproteobacteria* (90.9 ± 3.3%) during initial biofilm formation to dominance by *Deltaproteobacteria*, particularly *Geobacter* (68.7 ± 3.6%) in mature, electricity-generating anodes. Community diversity in the intermediate stage, just after robust current generation began, was double that at the early stage and nearly double that of mature anode communities. Maximum current densities at the intermediate stage, however, were relatively similar (~ 83%) to those achieved by mature-stage biofilms. Meta-proteomic analysis, correlated with population changes, revealed significant enrichment of categories specific to membrane and transport functions among proteins from electricity-producing biofilms. Proteins detected only in electricity-producing biofilms were associated with gluconeogenesis, the glyoxylate cycle, and fatty acid β-oxidation, as well as with denitrification and competitive inhibition.

**Conclusions:**

The results demonstrate that it is possible for an MFC microbial community to generate robust current densities while exhibiting high taxonomic diversity. Moreover, these data provide evidence to suggest that startup growth of air–cathode MFCs under conditions that promote the establishment of aerobic–anaerobic syntrophy may decrease startup times. This study represents the first investigation into protein expression of a complex BES anode biofilm community as a whole. The findings contribute to understanding of the molecular mechanisms at work during BES startup and suggest options for improvement of BES generation of bioelectricity from renewable biomass.

**Electronic supplementary material:**

The online version of this article (10.1186/s13068-018-1111-2) contains supplementary material, which is available to authorized users.

## Background

In bioelectrochemical systems (BESs), electrochemically active microbes convey electrons to or from a conductive electrode [1]. In some BES systems, such as microbial fuel cells (MFCs), bacteria living in a biofilm use the anode as an electron acceptor for electrons harvested from organic materials such as lignocellulosic biomass or waste byproducts, and the resulting current is harnessed to recover energy during wastewater treatment [2]. Other BES applications include removal of nutrients [3] or metals [4], desalination [5], and generation of bioproducts such as H_2_ [6], H_2_O_2_ [7], or organic molecules from CO_2_ and sunlight [8]. Successful commercial application of BES technologies will require increases in current generation and efficiency [9]. More detailed information regarding the fundamental mechanisms that enable bioelectricity generation will inform strategies for scale-up and new applications of BES technology [10].

Descriptions of the mechanisms behind electricity generation in BESs have relied primarily on model BES genera. Current generation mechanisms described for model BES genera like *Shewanella* and *Geobacter* include indirect electron transfer via soluble redox compounds [11], and direct electron transfer via outer membrane cytochromes [12] or pilus-like “nanowires” [13]. These discoveries have informed improvements in BES performance through design of electrode architecture [14], identification of limiting factors during electron transfer [15], and attempts at metabolic engineering of microbes or defined community [16]. Few studies, however, have attempted to determine which of these current generation mechanisms are most prevalent in mixed culture BES communities or how the interactions between members of BES consortia affect electricity generation. So far, those interactions primarily have been described in terms of community composition, quantified as relative abundance of 16S rRNA genes [17, 18]. This kind of ecological approach describes compositional changes of BES community in response to operational changes such as the type of substrate [19]. Metabolic syntrophies among BES community members are thought to explain the generally superior performance of a mixed community compared with pure cultures [20]. However, 16S rRNA profiling is not well suited to provide insights into interactions among community members. Identification of the community interactions and mechanisms of current generation at work during BES biofilm development may suggest strategies to reduce reactor startup time or increase community resilience to perturbation, thereby reducing operating costs and supporting commercial scale-up.

Appropriate tools for molecular investigations of microbial community function have emerged only recently [21]. Proteomics, in particular, has been useful for profiling protein expression of defined or undefined microbial communities [22]. These “meta-proteomics” studies have identified metabolic mechanisms behind multispecies fermentation [23], methanogenesis [24], or community response to a toxic perturbation [25]. A few studies have used proteomics methods to investigate electricity generation by model species or isolates [26, 27]. Meta-proteomics examinations of BES systems, however, have been limited to a single investigation of protein expression of biocathode biofilm organisms under optimal and suboptimal conditions [28]. This study identified several biocathode proteins associated with an optimal reactor potential for use of an electrode as an electron donor to fix CO_2_. The question of the proteins associated with anode mixed-species biofilms that generate electricity has not yet been addressed.

The goal of this study was to characterize protein expression that is distinctive to a MFC anode community when it is generating electricity. Specifically, a label-free meta-proteomics approach was used to compare protein expression in acetate-fed MFC anode biofilms before and after the onset of robust current generation. Since the types and abundances of proteins expressed depend on the types and abundances of microbial genera present in the community, we quantified changes in MFC community structure across developmental stages in terms of relative abundance of operational taxonomical units (OTUs). This information about community structure was integrated with meta-proteomics results in two ways. First, protein expression was normalized to abundance levels of individual genera during significance testing for differential expression of proteins. Second, OTU quantification was used as a method of orthogonal “validation” of meta-proteomics results by comparing relative abundance of genera based on OTUs with that based on genera associated with protein identifications. Compared with using a single method, this sort of mixed meta-omics approach offers the possibility of obtaining a more complete picture of the activities and interactions of the anode community members during MFC startup and electricity production. Such a picture may prove useful for improving MFC performance through reactor design, operating conditions, or community structure modification.

## Methods

### MFC setup, operation, and harvest

The single-chamber, membrane-free, air–cathode MFC design used in this study was similar to a previous design [29] and is described in detail in Additional file 1. The liquid volume of each MFC was 30 mL, and the area of the anode and air–cathode was 7.0 cm^2^. The MFCs were autoclaved prior to inoculation with a mixed culture inoculum that was derived from anaerobic digester sludge (Drake Water Reclamation Facility, Fort Collins, CO). The MFCs were fed 30 mM acetate by full batch replacement with a minimal medium described previously [29]. No exogenous redox mediators were used. MFCs were operated at room temperature with a 1 kΩ external resistor completing the anode–cathode circuit. Anodes from three replicate MFCs were harvested for each of four developmental stages: (i) bulk MFC suspension; (ii) early anode biofilm; (iii) intermediate anode biofilm; (iv) mature anode biofilm. Bulk suspension samples were collected 24 h after inoculation for Stage (i). Stage (ii) MFCs were harvested when they first reached current densities of  ~ 0.05 A/m^2^, an order of magnitude lower than that in Stage (iii) (~ 0.6 A/m^2^). Stage (iv) was characterized by higher current densities (0.7–0.8 A/m^2^) in repeated batches over a 2-year period.

## 16S rRNA gene sequencing and OTU analysis

Total DNA was extracted from biofilm scraped from each MFC anode using a Powersoil DNA Isolation Kit (MoBio Laboratories, Inc., Carlsbad, CA, USA), according to the manufacturer’s instructions. The V3–V7 region of 16S rRNA genes was sequenced at RTL Genomics (Lubbock, TX) using an Illumina MiSeq platform. Statistical comparison of the relative abundance of operational taxonomic units (OTUs) between communities from the four developmental stages was conducted with a nonparametric multivariate analysis of variance (npMANOVA) test, using the *vegan* package in R [30], as described in Additional file 1. For post hoc analysis, pairwise Pearson’s *r* correlations between consecutive pairs of anodes of each developmental condition were conducted using the *cor()* function in R and plotted using the *ggplot()* function. Pairwise correlations were investigated for significant differences between comparison type (e.g., early–intermediate correlations vs. early–mature correlations) by Tukey’s HSD test. In addition, a nonmetric multidimensional scaling (NMDS) plot was generated to compare clustering for each sample type as described in Additional file 1. Based on the results of the npMANOVA, Pearson’s correlations, and NMDS results, a similarity percentage (SIMPER) analysis [31] was conducted on the early and mature anode biofilms. This analysis quantifies the average contribution of each taxon to the overall difference between communities. Simpson’s Diversity Index (SDI) values were computed for each anode biofilm and compared for statistically significant differences between developmental stages with a Welch’s *t* test.

### Proteomic analysis

#### Protein extraction from MFC samples

A protein extraction method was developed specifically for anode biofilms. Briefly, biofilm scraped from each harvested anode was sonicated in lysis buffer (50 mM ammonium bicarbonate, 1% sodium deoxycholate, pH 8.2) for 5 min, subjected to a freeze–thaw cycle, and sonicated again. Sodium deoxycholate was chosen as a detergent to maximize unbiased protein recovery, including from membrane proteins [32]. Supernatant containing suspended proteins was collected (14,000×*g* for 20 min). Proteins were precipitated, quantified, and trypsin-digested according to standard methods, as described in Additional file 1.

#### LC–MS/MS analysis

Two micrograms of resuspended peptides were loaded onto a C18 trap (200 µm ID, 0.5 mm length, 120 Å, Eksigent Technologies) on the front end of a NanoLC 400 (Eksigent Technologies, Redwood City, CA, USA). A 2–80% gradient of acetonitrile (ACN) with 0.1% formic acid was used to elute the peptides (C18, 75 µm ID, 150 mm length, 120 Å, Eksigent Technologies, Redwood City, CA, USA) at a flow rate of 300 nL/min. Peptides were eluted (150 min) into the electrospray ionization chamber of a TripleTOF 5600 Q-TOF mass spectrometer (ABSciex, Redwood City, CA, USA) and ionized at 80 V. Up to 50 MS^2^ scans followed each MS^1^ scan, according to the order of intensity. Three technical replicate LC–MS/MS injections were performed for each biological replicate MFC anode. The MS proteomics data were deposited at the open access library of ProteomeXchange Consortium (http://www.proteomexchange.org/) [33].

#### Label-free quantification, statistical analysis, and metabolic interpretation of protein data

**Fig. 1:**
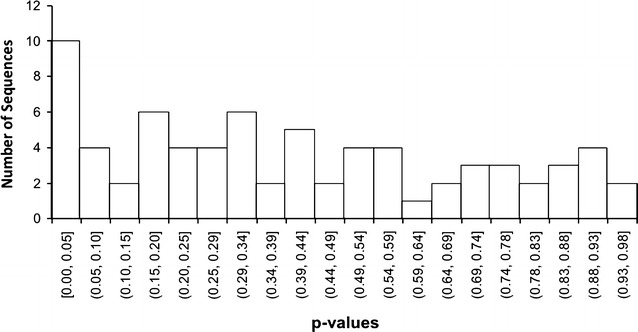
Histogram of all *p*-values from a Student’s *t* test (assuming equal variances) comparing modified NSAF values for proteins identified in common between early and intermediate replicate anode biofilms. X-axis values [a,b] indicate the range of *p*-values for each bin

Proteins were identified from LC–MS/MS spectra (ProteinPilot v.4.5 beta) using a fasta database consisting of the entire bacterial metaproteome (proteome filter “taxonomy: bacteria (2),” downloaded from Uniprot 2-15-15). The false discovery rate (FDR) was computed by ProteinPilot with a reversed-sequence decoy database using a threshold ≤ 0.01. For each protein, a modified version of the distributed normalized spectral abundance factor (NSAF) [34] was computed with custom R scripts, using the ProteinPilot “unused score”. For proteins identified in both early and intermediate MFC samples, this NSAF value was normalized to OTU abundance of the corresponding genus for that protein, thus accounting for population shifts in the community. Since no proteins were identified as differentially expressed after applying a *q*-value multiple-testing correction [35], but the histogram of *p*-values showed a right skew (Fig. 1), we present instead proteins-of-interest (POIs) that had log_2_-fold-change greater than 1 or less than − 1 and *p* < 0.05 in a Student’s *t* test [36]. For proteins detected only in one of the anode developmental stages (“uniquely detected proteins,” UDPs), Fisher’s exact tests implemented in Blast2GO (v.3.1.0) identified Gene Ontology (GO) categories significantly enriched (FDR < 0.05) [37] among either the early or intermediate UDPs. Since this study was concerned with proteins that are distinctive to current generation in MFCs, subsequent analysis focused on POIs with log_2_-fold-change > 1 as well as UDPs in intermediate MFCs (UDPIs). Analysis of KEGG pathways enriched among UDPIs was conducted by GhostKOALA, an automated metagenome annotation server that characterizes gene functions and pathways based on KEGG Orthology sequence assignments [38]. As for OTUs, the diversity of genera in early and intermediate communities was quantified by SDI on the relative abundance values of proteins identified by GhostKOALA, across replicate anode samples. Diversity in early or intermediate stages was compared by a two-tailed Welch’s *t* test on SDI values. A detailed description of protein identification, spectral count quantification, and statistical comparison is provided in Additional file 1.

### Scanning electron microscopy (SEM)

Sections (3 cm × 3 cm) of harvested anodes were removed using a sterile razor blade. Biofilms were fixed to the anode surface with an aqueous solution of 1.5% formaldehyde and 2.5% glutaraldehyde for 2 h at room temperature. Samples were washed with phosphate buffer and then sequentially in 60, 70, 80, 90, and 100% aqueous solutions of ethanol. Residual ethanol was evaporated, and samples were visualized on a Hitachi TM3000 SEM (Schaumburg, IL, USA).

## Results

### Current generation and MFC anode colonization

**Fig. 2:**
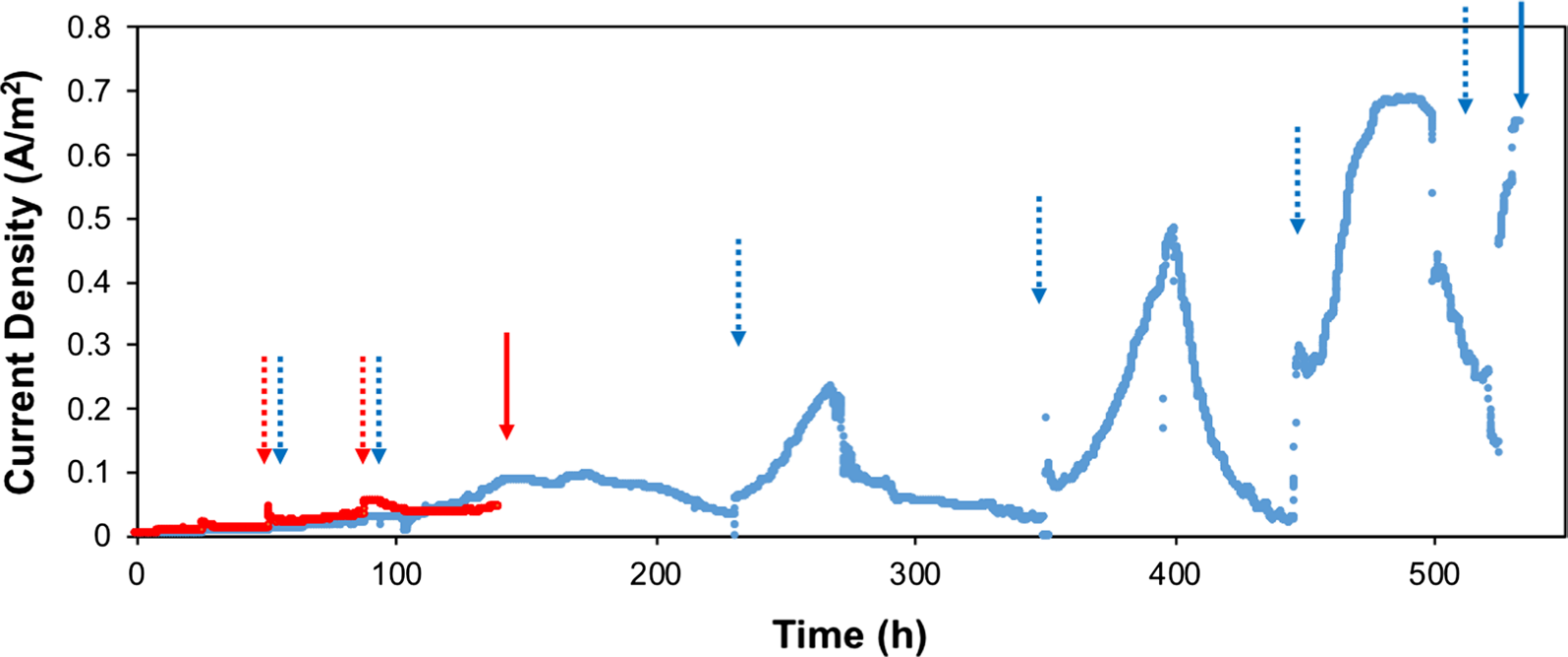
Representative current density of air–cathode MFCs that were compared with proteomics. Current density rose due to partial or complete replacement of medium. Dashed arrows represent full batch replacement of all medium in the MFC. Solid arrows indicate harvest point. Early anodes (red) were harvested 130.3 ± 9.1 h after inoculation, while intermediate anodes (blue) were harvested 523.7 ± 35.0 h after inoculation. Mature MFC performance data are shown in Additional file 2: Figure S3

The purpose of this study was to identify protein expression that is unique to MFC anode biofilm communities during electricity generation. Therefore, MFC anodes were harvested at different developmental stages before and after robust current generation was detected. The developmental stage of MFC biofilms was identified by current densities at the harvest point: 0.056 ± 0.005, 0.632 ± 0.059, and 0.764 ± 0.035 A/m^2^ for early, intermediate, and mature MFC biofilms, respectively. Early MFC biofilms were harvested after 130.3 ± 9.1 h of operation, at very low levels of electricity generation (Fig. 2). Initial adhesion of cells to anode fibers was observable at this stage, compared to unused carbon cloth (Additional file 2: Figure S1A and B). Intermediate MFCs were harvested after 523.7 ± 35.0 h, when current density increased to 0.6 A/m^2^. More extensive cell growth and biofilm structures were observed on intermediate anodes, compared to early anodes (Additional file 2: Figure S1C). Current densities and dynamics for replicate MFCs at the early and intermediate times were similar (Additional file 2: Figure S2). Mature anode biofilms were harvested after they generated maximum current densities of 0.7–0.8 A/m^2^ for more than 2 years (> 17,000 h) (Additional file 2: Figure S3). Several layers of cells surrounded by a thick extracellular matrix were observed on mature anodes (Additional file 2: Figure S1D).

### MFC biofilm community structure based on 16S rRNA gene amplicons and proteins

**Fig. 3:**
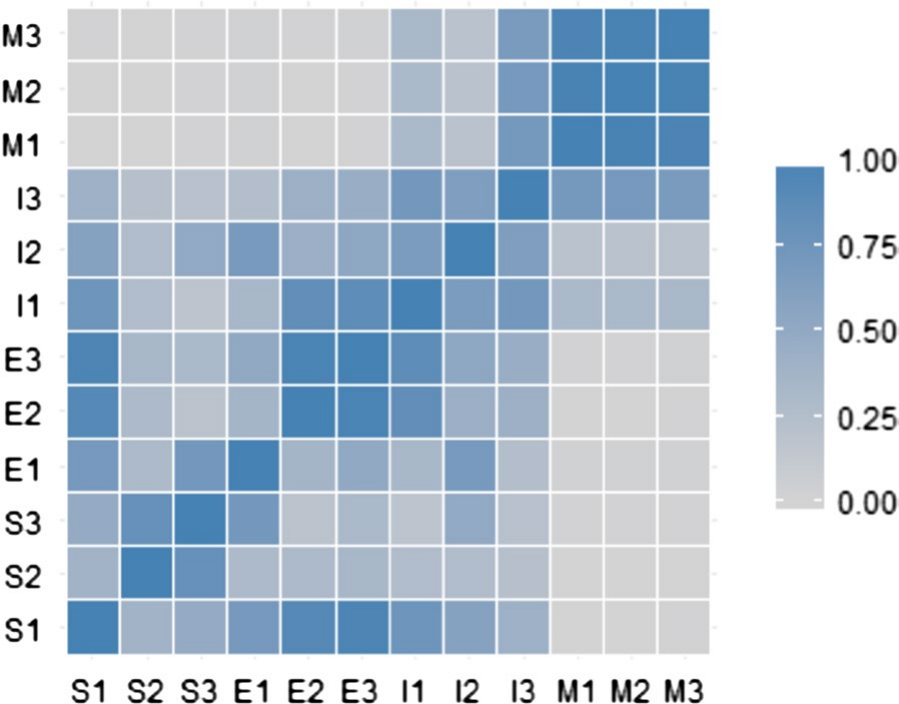
Matrix of Pearson’s correlations from pairwise comparisons between consecutive pairs of samples from the bulk solution (S), early anode (E), intermediate anode (I), and mature anode (M), with respect to 392 different OTUs. As specified by the legend, darker colors indicate a higher Pearson’s coefficient and thus more similarity between the two samples compared. A one-way ANOVA on Pearson’s coefficients confirmed that the MFC developmental stages were significantly different (*p* < 2 × 10^−9^)

Significant differences (*p* < 0.001, npMANOVA) in community composition as measured by OTUs were observed among the four MFC developmental stages (Additional file 3: Table S1). As a post hoc test, pairwise Pearson’s r correlations were calculated between consecutive pairs of biological replicates of each developmental stage. The correlation was lowest between the mature anode biofilm OTUs and OTUs from either the bulk solution (Pearson’s *r* = 0.005 ± 0.004) or early anode communities (Pearson’s *r* = 0.011 ± 0.007) (Fig. 3; Additional file 3: Table S1). The greatest degree of correlation was found between early and solution communities (*r* = 0.52 ± 0.30) and early and intermediate communities (*r* = 0.53 ± 0.22). The latter correlation between early and intermediate communities was significantly (*p* < 0.05, Tukey’s HSD) better than that between early and mature biofilms (*r* = 0.01 ± 0.01), consistent with the emergence of the intermediate biofilm community from the early community. Interestingly, the taxonomic diversity of the intermediate biofilm was significantly (*p* < 0.005, Welch’s *t* test) much greater than that of any of the other stages, exhibiting a SDI value more than twice that of the early biofilm (Table 1). The increase in diversity likely contributed to the lower degree of clustering of intermediate anode biofilm samples in the NMDS plot, compared to the other sample types (Additional file 2: Figure S4).

**Table 1:** Comparison of Simpson’s Diversity Index values for MFC communities at different developmental stages. The enumeration method refers to sequenced 16S rRNA gene amplicons (OTUs) or LC–MS/MS-identified proteins assigned to taxonomical groups by GhostKOALA. The error term is the standard deviation across microbial consortia for three independent replicate MFC anodes. GhostKOALA protein information for solution and mature biofilm communities is not available (NA) since proteomics analysis was not conducted on those samples

Enumeration method	Solution	Early	Intermediate	Mature
OTUs	0.28 ± 0.13	0.30 ± 0.12	0.85 ± 0.03	0.50 ± 0.05
GhostKOALA proteins	NA	0.43 ± 0.07	0.80 ± 0.01	NA

Since the early and mature communities were significantly different according to both npMANOVA and Pearson’s *r* correlation, SIMPER analysis was conducted to determine the percent contribution of individual taxa to the dissimilarity between those two communities. This analysis identified OTUs 132 (*Acinetobacter*), 333 (*Geobacter*), and 96 (*Pseudomonas*) contributing most to the differences between early and mature biofilm communities, with SIMPER cumulative contribution scores of 0.77, 0.41, and 0.63, respectively. This result from SIMPER analysis was corroborated by the decreasing relative abundance of total *Gammaproteobacteria* (*Acinetobacter*, *Pseudomonas*) and increasing *Deltaproteobacteria* (*Geobacter*) as the biofilms progressed from early to mature stages (Additional file 3: Tables S2 and S3). Moreover, the shift in dominance across the developmental stages between those three genera can be seen clearly in relative abundances of prominent OTUs (relative abundance greater than or equal to 1.0%) across developmental stages. Nearly all of the relative abundance attributed to each genus was due to the same OTUs (Additional file 3: Tables S3 and S4). For example, across all developmental stages nearly all of the relative abundance of *Geobacter* was due to OTU333, which had no identified species. Over the four developmental stages sampled, the number of OTUs with relative abundance greater than or equal to 1.0% decreased from seven (solution) to six (early biofilm), then increased to 17 OTUs (intermediate biofilm) before decreasing to just four OTUs in the mature biofilms. Genera that emerged to more than 1.0% in the intermediate biofilm included *Thauera* (OTU198), *Alcaligenes* (OTU288), *Geobacter* (OTU333), and an unknown *Synergistales* (OTU204). Of these, only the latter two OTUs were prominent in the mature biofilm; together with an unknown bacterium and an *Actinomyces*, they comprised over 86% of the mature community.

The relatively high degree of overall correlation between the early and solution communities (Additional file 3: Table S1) as well as the dominance of both early and solution communities by *Gammaproteobacteria*—i.e., *Acinetobacter* (OTU132) and *Pseudomonas* (OTUs 356 and 96) (Additional file 3: Tables S2, S3, S4)—suggested that those genera were early colonizers of the anode. Interestingly, *Pseudomonas* OTU356 was the most dominant OTU in the solution community but was only present at ~ 1.0% in the early biofilm community (Additional file 3: Table S4). In contrast, relative abundance of *Pseudomonas* OTU96 increased in abundance in the early biofilm compared to the solution community, suggesting that the species of *Pseudomonas* represented by OTU356 did not attach to the anode effectively.

**Fig. 4:**
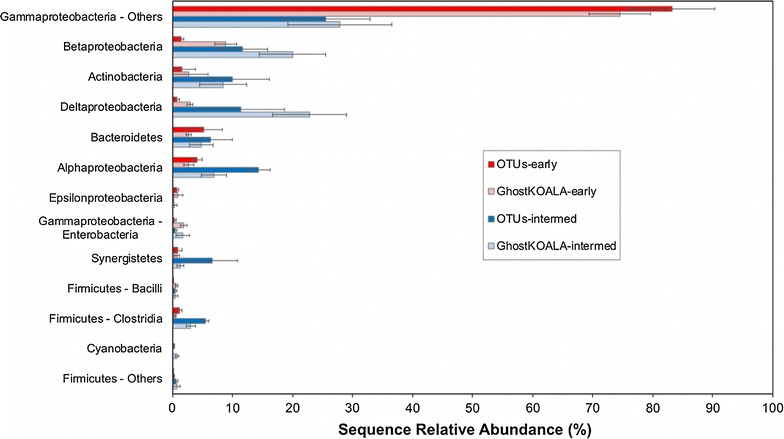
Relative abundances of genera associated with OTUs from 16S rRNA gene sequencing or GhostKOALA protein categorization. Relative abundances are from OTUs of early anode biofilms (dark red), GhostKOALA of early anode biofilms (light red), OTUs of intermediate biofilms (dark blue), and GhostKOALA of intermediate anode biofilms (light blue). Error bars represent standard deviations across samples from three independent biological replicate MFC anode biofilms. Only genera with relative abundance greater than 0.5% for at least one sample type are shown; a table of full relative abundance values is available in Additional file 3: Table S6

The GhostKOALA and OTU datasets were significantly correlated (adjusted *R*^2^ = 0.914, *p* < 2.2e^−16^) with regard to taxonomical relative abundance (Additional file 2: Figures S5 and S6). The taxonomies of OTUs and proteins both showed dominance of early anode biofilm communities by nonenteric *Gammaproteobacteria*, with increased relative abundance in intermediate biofilms for *Alpha*-, *Beta*-, and *Deltaproteobacteria*, as well as for *Actinobacteria*, *Synergistetes*, and *Firmicutes* (*Clostridia*) (Fig. 4). Moreover, OTU and GhostKOALA protein methods agreed that the taxonomic diversity of the intermediate MFCs was significantly (*p* < 0.005, Welch’s *t* test) greater than that of the early MFCs. With respect to diversity, SDI values based on GhostKOALA protein identifications (Additional file 1: Section G) were 0.43 ± 0.07 and 0.80 ± 0.01 for early and intermediate anode samples, respectively (Table 1). There was no significant difference between the two methods in SDI values for either the early (*p* > 0.2, Welch’s *t* test) or intermediate communities (*p* > 0.1, Welch’s *t* test). The broad phylogenetic agreement between the two methods with respect to genera identifications suggested that a representative extraction of proteins across the set of different members of the MFC community had been achieved.

A complete list of all 392 OTUs identified in this study, including taxonomy and abundance in each MFC sample, is provided in Additional file 4.

### Proteomics results

#### Summary of proteomics data features

The purpose of this study was to describe distinctive protein expression in MFC anode biofilm communities that are generating electricity compared to those that are not. Ideally, samples for this proteomic comparison would come from MFC biofilms that were identical in community composition and differed only with respect to current generation. In that case, any differences between the two meta-proteomes would be attributable to current generation, rather than to differences in community composition. While not identical in community composition, (see “MFC biofilm community structure based on 16S rRNA gene amplicons and proteins” section) the early and intermediate biofilms were more similar than the early and mature biofilms (Fig. 3; Additional file 3: Table S1). However, the intermediate anodes generated current densities nearly as high (~ 83%) as those generated by mature anodes. Therefore, we considered a comparison of the early and intermediate conditions to be the most reasonable approach to compare differences in anode biofilm protein expression with and without electricity production, while limiting as much as possible the extent to which those differences are just due to different community composition.

Across all LC–MS/MS samples for early and intermediate MFC biofilms, 8557 protein identifications were made at 1% FDR (5932 proteins identified by more than one peptide), resulting in 3866 nonredundant identifications across technical replicates. Additional information on the confidence of sequence identification for peptides and proteins is available in Additional Files 5 and 6, respectively. Across replicate anode biofilm samples, 1430 early and 1194 intermediate nonredundant proteins were identified (Additional file 2: Figure S7). Of the 853 proteins identified in at least one technical replicate of at least two anodes in a condition, 377 proteins were identified only in early anode biofilms, 182 proteins were identified only in intermediate anodes, and 87 proteins were identified in both early and intermediate anodes (Additional file 2: Figure S8). With a *q*-value multiple testing correction, none of these 87 proteins was identified as differentially expressed. However, since the histogram of *p*-values generally was skewed right (Fig. 1), the seven proteins with *p* < 0.05 and log_2_-fold-change greater than 1 or less than − 1 are presented as POIs between the two conditions (Table 2) [36]. Five of those POIs had a log_2_-fold-change greater than 1, indicating greater abundance in the intermediate compared with the early biofilms. Therefore, a total of 187 proteins were determined to be either a UDPI (UDP detected only in intermediate anode biofilms) or a POI being more abundant in the intermediate biofilms. Subsequent metabolic analysis focused on these 187 proteins as representatives of changes in the proteome most distinctively associated with the onset of electricity production.

**Table 2:** Proteins of interest that were shared in common between early and intermediate MFC biofilms. These proteins met a criterion of *p* < 0.05 (Student’s *t* test, no multiple testing correction) with log2-fold-change (log2 FC) of either > 1 or < − 1 for a ratio of intermediate/early conditions

Uniprot ID	Protein name	Genus	*p* value	log_2_FC (intermed/early)
A0A073KKY5	Porin	*Shewanella*	0.016	3.2
A0A077F4W8	Aromatic hydrocarbon degradation protein	*Pseudomonas*	0.007	2.7
A0A077F872	Outer membrane protein H1	*Pseudomonas*	0.007	2.1
A0A067A3Q5	Outer membrane insertion C-terminal signal domain protein	*Pseudomonas*	0.016	2.1
A0A075PCY9	Porin	*Pseudomonas*	0.04	2
A0A077F9B6	Polyribonucleotide nucleotidyltransferase	*Pseudomonas*	0.007	− 2.7
A0A066ZUQ4	Aldehyde dehydrogenase family protein	*Pseudomonas*	0.006	− 3.4

#### Significant enrichment of membrane and transport proteins

**Fig. 5:**
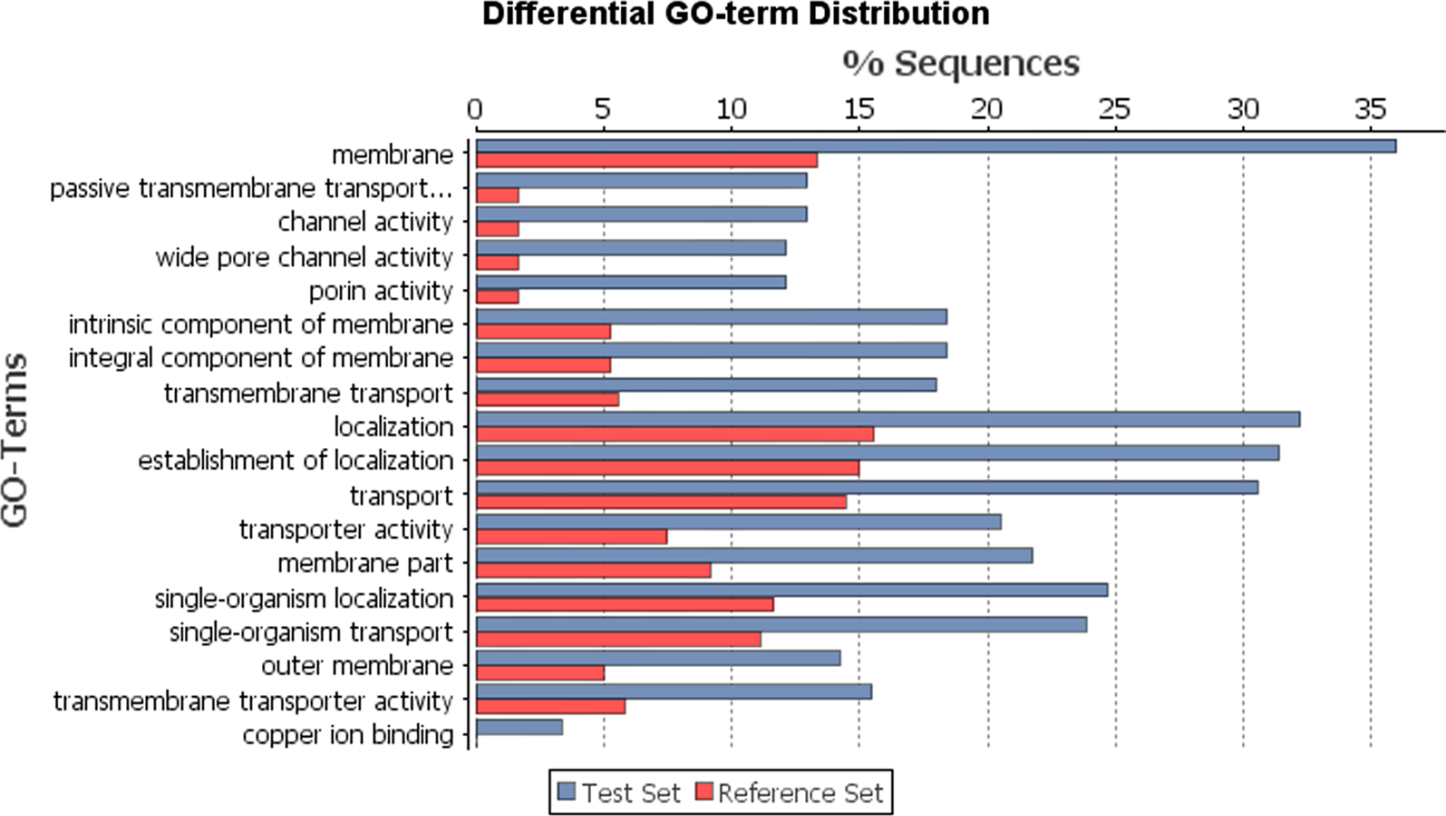
Percentages of intermediate (blue) and early (red) MFC biofilm protein sequences assigned to gene ontology (GO) categories. Significant differences in protein levels between intermediate and early conditions were determined by a Fisher’s exact test implemented in Blast2GO v. 3.1.0 with a Benjamini-Hochberg multiple testing correction factor (FDR < 0.05). A total of 733 sequences were assigned to GO categories

**Fig. 6:**
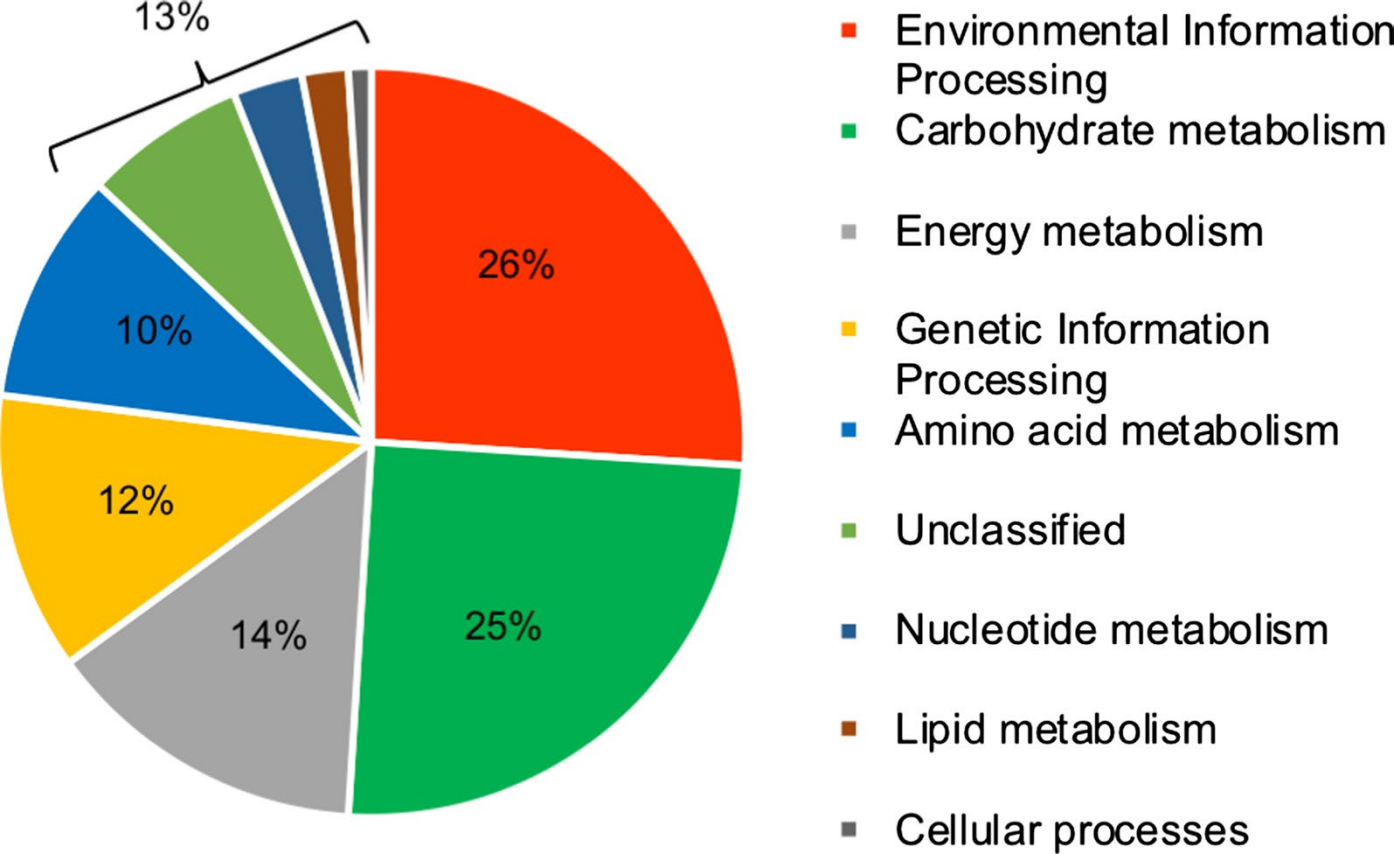
KEGG functional categorization of UDPIs and POIs that were more abundant in intermediate MFC biofilms. Proteins were identified and categorized using the GhostKOALA tool against the entire prokaryotic database

The GO groups gene products into interrelated categories associated with biological processes, cellular components, and molecular function. A Fisher’s exact test identified 18 GO functional categories that were significantly enriched among the set of proteins from intermediate anode biofilms, compared with early anode biofilms (Fig. 5). The most enriched GO category was “membrane”, which was associated with over 35% of the intermediate proteins but less than 14% of early proteins. Five additional enriched GO categories were explicitly related to membranes (e.g., “integral component of membrane” and “transmembrane transport”), and nearly all of the 12 remaining enriched GO categories among intermediate proteins were related either to transport or localization. Moreover, four of the five more abundant POIs (and neither of the less-abundant POIs) were associated with membrane processes or transport (Table 2). KEGG analysis by GhostKOALA corroborated this enrichment in membrane and transport processes in the intermediate anode biofilms. Of the 134 UDPIs that were identified with KEGG annotations, 35 proteins (26%) fell into the category of “environmental information processing,” making it the most-abundant KEGG category represented among UDPIs (Fig. 6). This category includes subcategories of membrane transport, signal transduction, and signaling molecule interactions (Additional file 3: Table S5). In addition, UDPIs involved in fatty acid biosynthesis were an especially abundant type of protein related to membranes—in this case associated with forming the membranes themselves (Additional file 3: Table S5).

Several of the membrane UDPIs have been explicitly associated with electricity production. The 63 *Geobacter* UDPIs included several cytochromes, including OmcX, a lipoprotein c-type outer membrane cytochrome (OMC) that has been shown to be necessary for current generation [39] (Additional file 3: Table S5). In addition, OmcS, a pili-associated cytochrome [40] was detected in both conditions and was more abundant in intermediate than early anodes, although not significantly so. No other OMC proteins were identified, which was unexpected considering that membrane proteins generally were significantly enriched among UDPIs, and OMC proteins have been shown to be critical for electron transfer to the anode by some genera, including *Geobacter* [41]. The lack of detected *Geobacter* OMC proteins suggests that it used a different mechanism for electron transfer during these initial stages of current generation.

#### Central carbon metabolism

Central metabolic pathways represented among UDPIs included the TCA cycle, fatty acid β-oxidation, fatty acid biosynthesis, and acetate uptake and activation (Additional file 3: Table S5). Anaerobic central metabolism was represented by Por and Kor enzymes, the anaerobic analogs to pyruvate dehydrogenase and α-ketoglutarate dehydrogenase, respectively [42, 43]. Carbohydrate metabolism was the second most abundant KEGG category represented among UDPIs (Fig. 6), despite the lack of carbohydrates in the medium. These proteins likely were participating in gluconeogenesis; enzymes were found among UDPIs that covered the gluconeogenesis pathway from oxaloacetate to glyceraldehyde-3-phosphate. In addition, isocitrate lyase was a UDPI from *Thauera*, a facultative genus that comprised 5.9 ± 1.9% of the intermediate MFC anode community. This enzyme catalyzes the key step of the glyoxylate shunt, the anabolic cycle that converts a C_2_ compound like acetate/acetyl-CoA to C_4_ compounds for entry into gluconeogenesis [44].

#### Nitrogen metabolism

Glutamate dehydrogenase (GDH) was the most abundant UDPI in terms of relative abundance of OTUs in the intermediate biofilms. It was also the most well-represented UDPI across taxa, detected in five different genera (Additional file 3: Table S5). This enzyme stores or releases ammonia during amino acid synthesis; in strict anaerobes, GDH may act as an electron sink in association with aminotransferases, two of which also were identified for *Geobacter* [45]. A nitrogen-fixation scaffold protein NifU was one of seven *Geobacter* UDPIs that were associated with iron–sulfur cluster binding [46].

Evidence for both nitrification and denitrification was found among intermediate MFCs. The combination of ammonium as the supplied nitrogen source and microaerobic conditions provided a suitable environment for nitrification, as suggested by detection of *Nitrosomonas* OTUs. Evidence for denitrification included a UDPI for *Pseudomonas stutzeri*, a well-known denitrifier, as well as two UDPIs from genus *Nitratireductor* [47]. Additional denitrifying UDPIs included nitrite reductase, nitric oxide reductase, a nitrate-induced formate dehydrogenase, and three nitrous oxide reductases (NosZ) (Additional file 3: Table S5). One of the latter was from *Azoarcus*, a model genus for nitrogen fixation [48]. Another NosZ was one of 16 UDPIs from nitrifying-denitrifier *Alcaligenes*, a genus comprising approximately 3% of intermediate OTUs but less than 0.1% of early OTUs. The activity of NosZ suggests the presence of N_2_O, an intermediate formed during both nitrification and denitrification [49].

#### *Geobacter* interactions within MFC biofilms

With 63 UDPIs, *Geobacter* was the most abundant genus represented among UDPIs (*Pseudomonas* was second most abundant, with 19 UDPIs). The flagellar UDPI FliC suggested active participation of *Geobacter* in formation of mixed culture biofilm structures [50]. Another *Geobacter* UDPI, cysteine synthase A, is known to produce a toxin that inhibits growth and biofilm production of neighboring bacteria [51]. Expression of cysteine synthase A suggests that *Geobacter* uses methods of antagonism to suppress the growth of nearby competitors, in addition to outcompeting them with superior capacities for anaerobic respiration of the anode. A *Geobacter* phage tail sheath protein also was a UDPI. Phage proteins recently have been associated with increased growth and Fe(III) respiration by *Geobacter* in uranium-contaminated soils [52]. Finally, superoxide dismutase and rubredoxin:oxygen/nitric oxide oxidoreductase, scavengers of oxygen and nitrogen compounds, were *Geobacter* UPDIs [53, 54].

## Discussion

### Community dynamics during MFC biofilm development

The relative abundance of OTUs in the solution and early biofilm samples indicated that early colonizers of the anode included *Acinetobacter* and *Pseudomonas*, two genera generally considered aerobes, although some studies have shown electricity-producing capabilities for members of each [55, 56]. The dominance of aerobic or facultative genera in early developmental stages likely was due to the microaerobic conditions of air–cathode MFCs [29]. As the anode biofilm grew in structure and complexity (Additional file 2: Figure S1), these genera would have declined in relative abundance, while anaerobic genera such as *Geobacter* became more prevalent. For example, *Pseudomonas* was a prominent member of both the solution (~ 58%) and early biofilm samples (~ 55%) but decreased to ~ 18% by the intermediate stage and was scarce (< 1%) by the mature stage (Additional file 3: Table S3). Some aerobes persisted in the intermediate MFC community, however, possibly occupying a niche of oxygen consumption to maintain anoxic conditions for anode-reducing genera [57]. For example, *Alcaligenes*, an aerobe in Class *Betaproteobacteria*, increased in OTU relative abundance from 0.2 ± 0.2% in the early MFCs to 3.0 ± 2.1% in the intermediate MFCs. Nearly 9% of all UDPIs were from *Alcaligenes*, including proteins involved in the TCA cycle and endogenous peroxide scavenging, suggesting active metabolism by this genus. These results suggest that aerobes in air–cathode MFCs are important both for early colonization of the anode and for successful establishment of an electricity-producing community. Since the importance of oxygen during startup was not known prior to the start of these experiments, oxygen was not measured in these experiments. Future work, however, could modulate dissolved oxygen during MFC startup to determine how the presence of dissolved oxygen affects anode biofilm formation and the development of syntrophic relationships that provide oxygen-tolerance to the mature MFC community. This approach could be especially interesting using different inoculum sources, including those derived from aerobic wastewater that are known to result in more diverse final anode communities [58]. For more complex carbon sources than acetate, the role of aerobes or facultative organisms as oxygen-scavengers would need to be distinguished from their roles as degraders of complex substrates [59, 60].

The intermediate MFC achieved a maximum current density 83% of that generated by mature MFCs, despite substantially lower relative abundance of *Geobacter* (11.0 ± 7.1%) than the mature MFC community (68.7 ± 3.4%) (Additional file 3: Table S3). One possible explanation is that *Geobacter* need only to reach some threshold abundance level in the biofilm community for the MFC to generate high current densities. In that scenario, current densities continue to increase with additional enrichment of *Geobacter* but not at the same rate as below the threshold *Geobacter* abundance level, perhaps due to other limitations of the MFC reactor system such as internal resistance [61]. An alternative explanation is that other genera were responsible for some electricity generation. Indeed, several of the taxa enriched in the intermediate MFCs have been associated previously with electricity production, including *Actinobacteria* [62], *Alphaproteobacteria* [63], *Betaproteobacteria* [64], *Epsilonbacteria* [65], *Firmicutes* [18], *Bacteroidetes* [66], and *Synergistetes* [67]. The presence of these genera in the intermediate anode biofilms, along with the much greater diversity in the intermediate community compared with the mature community (Table 1), suggests the possibility of maintaining a diverse MFC community while still generating high current densities. Previous work has indicated that a diverse anode community with limited *Geobacter* can produce high current densities, but this occurred when treating complex wastewater with varying characteristics and endogenous organisms [68, 69] or when an aerobic wastewater source was used as inoculum [58]. Increased diversity can confer resilience to perturbations due to functional redundancy in the community structure [70]. Therefore, the results presented here suggest the possibility that engineering higher MFC community diversity—for example, through changes in inoculum source [58], carbon source [71], anode potential [72], or dissolved oxygen concentrations—could improve resilience without sacrificing MFC performance. Future work should investigate the effect of diversity per se on MFC current densities, startup time, and resilience to perturbations, perhaps by mixing a base inoculum high in *Geobacter* (e.g., effluent or biofilm material from a mature MFC running on acetate, as suggested previously [73]) with more diverse cultures from MFCs or other sources and comparing performance during degradation of influent streams of various complexity.

Comparison of the relative abundance of OTUs across biofilm developmental stages suggested that the intermediate biofilm represented a transitional state between early and mature biofilms. The intermediate community was better correlated to the early community than was the mature community (Additional file 3: Table S1), consistent with the emergence of the intermediate biofilm community from the early community. Moreover, a lack of correlation (Fig. 3) and reduced spatial clustering in a NMDS plot (Additional file 2: Figure S4) among intermediate biofilm replicate samples with respect to OTUs, may be explained by the high variability in the intermediate biofilms during a transitional stage. Only a few studies have investigated MFC community dynamics during startup. The increase in *Geobacter* relative abundance observed during transition from early (~ 6 days’ operation) to intermediate (~ 20 days’ operation) was consistent with a previous study using an acetate-fed, air–cathode MFC [74]. In the cited study, however, there was a clear decrease in *Bacillus* and little change in *Pseudomonas* relative abundance, while in the present study, *Bacillus* was nearly nondetectable in any sample and *Pseudomonas* clearly decreased. The difference likely was due to different inoculum sources (primary clarifier vs. anaerobic digester sludge). Interestingly, in both studies, *Rhizobiales* increased in relative abundance over the 6–20 day timescale. In the present work, the biofilm community continued to evolve after current density had achieved a high level, decreasing in diversity, as observed previously [63, 67, 75]. In the intermediate biofilm, there were 17 prominent OTUs that included a mixture of both aerobic (*Pseudomonas*, *Alcaligenes*, *Acinetobacter*, *Rhodococcus*) and anaerobic (*Geobacter*, *Synergistales*, *Clostridium*, *Rhizobiales*) taxa (Additional file 3: Table S4). In contrast, the mature biofilms were dominated by fewer than five genera, the most prominent of which by far was *Geobacter*, consistent with many previous examinations of anode communities [60, 71]. The second most abundant genus in the mature MFCs—an unknown member of *Bacteria*—may have been responsible for consuming residual oxygen to generate anaerobic conditions. The establishment of anoxic conditions is supported by the anode activity of *Geobacter* as well as by the persistence of *Synergistales*, an anaerobic, biogas producer that previously was identified as a member of a MFC “core microbiome” [67]. The reduction in number of OTUs in the mature anode biofilms suggests that MFC anode community development is competitive: many electrogenic genera attach to the anode but they are outcompeted (or actively suppressed, as suggested by proteomics results) by organisms with superior electricity generating capabilities, such as *Geobacter*.

The correlation of genera from OTUs with those from protein identifications (Table 1; Additional file 2: Figure S5) serves to corroborate the taxonomical part of the proteomics results. The proteomics results may be used, however, to gain additional taxonomical information about organisms identified by OTUs that was not provided by the OTU method. For example, in the intermediate biofilm, an unknown *Rhizobiales* (OTU380) is a prominent OTU, but sequencing did not provide more detailed taxonomical information. In the proteomics results for the intermediate biofilm, however, UDPIs were identified for *Nitratireductor*, a genus in Order *Rhizobiales*. Similarly, UDPIs were identified for *Mycobacterium* and *Synergistes*, members of Order *Actinomycetales* and Order *Synergistales*, respectively, each of which was a prominent OTU in the intermediate anodes (Additional file 3: Table S4). This use of taxonomical information from proteomics analysis suggests an additional way that proteomics results and OTU data from 16S rRNA gene sequencing may be integrated.

### Membrane proteins in *Geobacter* and other species

Membrane and transport proteins were significantly (Fisher’s exact test *p* < 0.05) more abundant in intermediate MFC biofilms, compared with early MFC biofilms (Fig. 5). Likewise, four of the five POIs more abundant in intermediate MFC biofilms were associated with membranes (Table 2). Membrane proteins contributing to structural integrity may be important during biofilm maturation. Moreover, mechanisms of interspecies chemical signaling through membrane channels [76] may play an important role in the development of effective electricity-generating MFC biofilms, and warrants additional study.

Membrane UDPIs for *Geobacter* included those involved in the steps of electron transfer in anaerobic respiration: NADH dehydrogenase, inner membrane *cb* cytochrome complex, ResB-like cytochromes, and OMC proteins (Additional file 3: Table S5). OMC proteins—outer membrane, c-type cytochromes containing a CXXCH motif—are responsible for exocellular transfer of electrons out of the cell to acceptors such as Fe(III) or an electrode [67]. Only two OMC proteins were detected in early and intermediate anode samples. OmcS, located along conductive type IV pili [77], was identified here in both early and intermediate MFCs, but OmcS was not a POI after normalization to *Geobacter* OTUs. Expression of OmcS has been shown to be independent of expression of other OMC proteins [78]; nevertheless, the lack of detected *Geobacter* OMC proteins was unexpected, since OMC proteins generally are associated with current generation by this genus [12]. It is possible that OMC proteins were simply not recovered during protein extraction, although this would be surprising, considering the enrichment of membrane proteins overall among UDPIs. Alternatively, considering that a dearth of OMC protein expression does not preclude completely current generation [79], it is possible that alternative methods of electron transfer were being used by *Geobacter* during these initial stages of robust current generation [80]. Competition with other species in the MFC biofilm may have altered patterns of OMC expression by *Geobacter*. In fact, the lack of detected OMC proteins may be consistent with the hypothesis that other genera in the community were responsible for electricity generation in these intermediate MFCs (“Community dynamics during MFC biofilm development” section). Additional proteomic studies comparing *Geobacter* protein expression during electricity production as part of a community with that in pure culture would shed light on important interactions that affect initial stages of electricity production.

### Gluconeogenesis and fatty acid metabolism by *Geobacter* and *Thauera*

As biofilms grew in intermediate MFCs, carbohydrate requirements for exopolysaccharide (EPS) production would have increased (Additional file 2: Figure S1). Since acetate was the sole carbon source available, the sugars used to produce EPS must have been synthesized through gluconeogenesis. In *Geobacter*, the UDPI succinyl:acetate coenzyme A transferase converts acetate to acetyl-CoA, the substrate for the Por enzyme (also a UDPI) to synthesize pyruvate *en route* to phosphoenolpyruvate (PEP) and then to 3-phosphoglycerate. The latter is the substrate for phosphoglycerate kinase, another *Geobacter* UDPI that is associated with gluconeogenesis.

In contrast to *Geobacter*, gluconeogenesis in *Thauera* was fed by the glyoxylate cycle, as indicated by isocitrate lyase and PEP carboxykinase as UDPIs. A previous meta-proteomics study identified the glyoxylate cycle as an upregulated process during the aerobic phase of enhanced biological phosphate removal by wastewater community [81]. *Thauera*, a facultative genus found in wastewater, is known for production of abundant EPS [82]. The gluconeogenesis activity observed here suggests that *Thauera* was actively involved in building biofilm in intermediate MFC anodes, perhaps as an aerobic syntrophic partner of *Geobacter*. Therefore, operating MFCs under conditions favorable for *Thauera* (i.e., microaerobically, with aromatic compounds as carbon source) could encourage such syntrophy and biofilm building, promoting faster initial biofilm establishment on the anode during MFC startup.

When carbohydrates are limited, β-oxidation of fatty acids provides acetyl-CoA that enters the TCA/glyoxylate cycle [83]. The UDPI evidence for β-oxidation was surprising here, since the 30 mM acetate in the MFC medium would have been expected to provide sufficient acetyl-CoA for both energy-generation and anabolic processes. For *Geobacter*, β-oxidation may have been coupled with the activity of Kor [41]. The products of β-oxidation also may have been used for biosynthesis of new fatty acids through the FASII system [84]; *Geobacter* and *Thauera* UDPIs of this system were found. An enhanced rate of fatty acid turnover would be consistent with the overall enrichment of membrane proteins in intermediate stage MFC biofilms as cytochromes or transport proteins are inserted into the lipid bilayer. Thus, the proteomics results presented here suggest that adding medium components that promote fatty acid biosynthesis could assist anode biofilm development during startup [85].

### Interactions in MFC biofilms

Interactions between organisms in a multispecies biofilm are critical for the emergence of higher-order biofilm properties such as functional stability [86]. Recent advances in omics methods have revealed coordinated interactions between cells in complex biofilms [87–89]. Here, antagonism by *Geobacter* toward other biofilm genera was suggested by the UDPI cysteine synthase A. This protein is part of a contact-dependent inhibition system, a direct cell-to-cell interaction mechanism used by some species for broad-spectrum inhibition of biofilm formation by other species [90]. The intermediate anode biofilm clearly exhibited a rapid rise in diversity compared with the early biofilm (Table 1), possibly including genera that have electrogenic abilities. Increased competition at the intermediate stage may have elicited antagonistic protein expression by *Geobacter* to actively inhibit competitors in the biofilm. Few previous studies have investigated this sort of antagonism by *Geobacter* in mixed consortia or the extent to which the dominance of *Geobacter* in MFCs or other anaerobic communities may be due to active inhibition of other genera, in addition to the metabolic advantages conferred by its robust capabilities for anaerobic respiration. *Geobacter* may gain additional competitive advantages through flagellar chemotaxis to gain proximity to the anode surface. Flagella—such as FliC, a *Geobacter* UDPI in the present study—have been associated with biofilm proliferation [91] as well as with current generation [79] or dissimilatory respiration of iron oxides by *Geobacter* [92]. Future work could take a strain-resolved proteomics approach [93] to comparing protein expression in BESs by *Geobacter* in pure culture [27] or as a dominant member of a constrained community to that as part of a diverse consortium. Such an approach could identify differences in the role of *Geobacter* role or mechanisms of action when involved with different types of communities, and perhaps identify mechanisms of antagonism against competitors, especially during MFC startup.

Several UDPIs were associated with the production of, or response to, hydrogen peroxide, a product of aerobic metabolism that plays roles in cell signaling, competitive inhibition, or spatial differentiation in biofilms [94]. A superoxide dismutase UDPI suggests that *Geobacter* may be producing hydrogen peroxide, to which UDPIs from other genera (*Rhodococcus* and *Alcaligenes*) may have responded (Additional file 3: Table S5). In addition to enabling *Geobacter* to withstand microaerobic conditions, peroxide production may be a method of competitive inhibition to kill off cells from other genera, releasing DNA that can stimulate biofilm formation [95].

Finally, several UDPIs suggested a response to reactive nitrogenous species, particularly NO and N_2_O (Additional file 3: Table S4) [96]. In intermediate anodes, both nitrifiers and denitrifiers were found in low relative abundance, in contrast to the high relative abundance of OTUs (~ 3%) and UDPIs (~ 8.5%) for *Alcaligenes*. This nitrifying–denitrifying genus has been shown to convert ammonium to molecular N_2_ under aerobic conditions, generating N_2_O as a byproduct [97]. The generation of N_2_ from denitrification by *Alcaligenes* may have triggered expression of the nitrogen-fixation UDPI NifU in *Geobacter* [45]. Nitrification and/or denitrification may have two negative impacts on electricity generation in MFCs: reducing nitrogen availability for electricity-generating genera and providing nitrate as an electron acceptor that competes with the anode. Additional study of nitrogen metabolism during MFC biofilm community development may suggest methods to reduce these negative impacts, or, alternatively, assist recent efforts to apply BES technologies for nitrogen removal or recovery.

## Conclusions

This study is the first to investigate global protein expression distinctive to electricity generation by a mixed microbial community. Changes in protein expression and community structure as a MFC begins producing electricity inform fundamental understanding of anode biofilm function and strategies to improve MFC performance. The results presented here suggest that community composition during MFC anode biofilm development is dynamic, shifting from dominance by aerobic taxa in early developmental stages to dominance by *Geobacter*. During that transition, an intermediate stage generates robust current densities, but with considerably greater community diversity than mature MFCs. Initiation of electricity production was associated with enrichment in membrane proteins related to transport and electron transfer, although not in conductive pili. Proteins involved in central carbon metabolism such as the TCA/glyoxylate cycle and gluconeogenesis that were enriched during electricity production suggest EPS biosynthesis by *Geobacter* and *Thauera* genera. Interactions between members of the electricity-producing community were indicated by proteins responsible for the production and response to reactive compounds such as N_2_O. Competitive interactions between *Geobacter* and other MFC community members were suggested by a contact-dependent inhibition cysteine synthase A protein and a phage tail protein in *Geobacter*. The results presented in this study suggest strategies to manipulate anode community interactions—for example, promoting aerobic EPS formation during early biofilm development, or reducing nitrification and denitrification—that may reduce startup times and improve efficiency of electricity generation in air–cathode MFCs or other BES technologies. This kind of mechanistic information will be critical for scale-up and integration of BES applications into the landscape of new technologies generating bioenergy from renewable biomass.

## Additional files

**Additional file 1.** Additional method descriptions. (A) Construction and operation of air–cathode MFCs; (B) harvest of MFC anodes; (C) DNA extraction, amplification, and sequencing; (D) quantitation and analysis of 16S rRNA gene amplicons; (E) extraction and digestion of proteins from MFC anode biofilms; (F) protein identification, label-free quantification and statistical analysis; (G) metabolic pathways analysis with gene ontology and KEGG: and (H) comparison of phylogenies from DNA sequencing and metaproteomics.

**Additional file 2: Figure S1.** Scanning electron microscope images (400× or 3,000× magnification) of MFC anode carbon cloth fibers at (A) non-inoculated, (B) early, (C) intermediate, and (C) mature stages of biofilm development. **Figure S2.** Current density for replicates of early (top) and intermediate (bottom) MFCs (inoculum originally derived from anaerobic digester sludge) after enrichment on batches of 30 mM acetate. **Figure S3.** Current density of a mature MFC (inoculum originally derived from anaerobic digester sludge) after enrichment for over 2 years on batches of 30 mM acetate. **Figure S4.** Non-metric multidimensional scaling (NMDS) plot of MFC anode OTUs along two principal coordinates. OTUs were identified by MiSeq sequencing of MFC anode 16S rRNA gene amplicons from three biological replicates of four different developmental conditions: S (solution), E (early biofilm), I (intermediate biofilm), M (mature biofilm). In the R *vegan* package, a Gower dissimilarity matrix was constructed with the metaMDS function from a sample-by-species matrix of OTU counts. The ordiplot, orditorp, and ordihull functions then were used to generate the NMDS plot. Similarity in relative abundance of genera is represented as spatial proximity within the two principal coordinates. Samples for S and E were clustered close enough together to obscure distinction of sample replicates. **Figure S5.** Linear regression of OTUs from MiSeq sequencing of 16S rRNA gene amplicons (“otu”) against proteins identified by GhostKOALA (“koala”) with respect to relative abundance of taxa. The linear regression was performed in R using the *lm* function on paired relative abundance values for each taxon in each biological replicate anode biofilm, excluding the intercept. Adjusted R^2^ = 0.914, *p*-value < 2.2e^−16^. **Figure S6.** Residuals vs. fitted plots for linear regression of taxon relative abundances, as quantified by GhostKOALA identification of proteins (“koala”) and OTUs from MiSeq sequencing of 16S rRNA gene amplicons (“otu”). The ten pairwise comparisons with the greatest positive or negative residuals are labeled. **Figure S7.** Venn diagrams of numbers of protein identifications identified in and shared between three biological replicates of (A) early MFC anodes and (B) intermediate MFC anodes. **Figure S8.** Venn diagram of proteins identified in early and intermediate anode development stages. Proteins in the overlapping area were identified in both conditions in at least one technical replicate of at least two biological replicates. The upper section of the overlapping area represents DEPs significantly more abundant in the intermediate condition, while the lower section represents DEPs significantly more abundant in the early condition.

**Additional file 3: Table S1.** Comparison of OTU relative abundance between four MFC developmental conditions: early (E), intermediate (I), mature (M) and solution (S), as identified by MiSeq sequencing of 16S rRNA genes. Each developmental stage was represented by three independent biological replicate samples. A nonparametric MANOVA *p*-value was generated by a comparison between all conditions. As a post hoc test between conditions, each replicate sample was compared against each replicate sample in the other condition by Pearson’s pairwise correlation, thereby generating mean and standard deviation Pearsons’ r coefficients for each pairwise comparison. Coefficients with different subscript letters indicate Pearson’s r coefficients that differ significantly (Tukey’s HSD test, p < 0.05). **Table S2.** Relative abundance across developmental stages of OTUs contained within different classes of the phylum Proteobacteria. Error terms represent ± one standard deviation across three separate MFC anode (or solution) samples. **Table S3.** Relative abundance across developmental stages of the three genera responsible for the majority of significant differences between early and mature MFC anode communities. Error terms represent ± one standard deviation across three separate MFC anode (or solution) samples. **Table S4.** Prominent OTUs (relative abundance greater than or equal to 1.0%) in MFC communities at each stage of development. The OTU column shows the identification number for each particular OTU. Solution samples were from the bulk solution of the MFCs 24 h after inoculation. The column for %OTUs signifies the percentage of OTUs in that sample that were identified as the specified OTU. The error term represents ± one standard deviation (SD) across three biological replicate samples. **Table S5.** Selected UDPIs categorized by broad metabolic process, as determined by KEGG, GO, Uniprot, and literature review. **Table S6.** Complete relative abundance values (mean % OTUs in a sample) for taxa in common between GhostKOALA annotation of proteins and OTUs from MiSeq sequencing of 16S rRNA gene amplicons from early and intermediate MFC anode biofilm samples. Relative abundances of OTUs are also shown for the solution and mature biofilm samples.

**Additional file 4.** Table of 16S rRNA gene MiSeq OTUs.

**Additional file 5.** Peptide sequences and confidence values for early and intermediate biofilm samples.

**Additional file 6.** Uniprot accession IDs, protein names, confidence values, unused scores, and number of sequences identified with 95% confidence for each protein in early and intermediate MFC anode biofilms.
