# Supplementary material for: Meta-proteomic analysis of protein expression distinctive to electricity-generating biofilm communities in air-cathode microbial fuel cells
Source: Biotechnol Biofuels. 2018 Apr 23;11:121. doi: 10.1186/s13068-018-1111-2 (PMC5913794; doi:10.1186/s13068-018-1111-2)
Supplement: Supplementary file 1 — Additional file 1. Additional method descriptions. (A) Construction and operation of air–cathode MFCs; (B) harvest of MFC anodes; (C) DNA extraction, amplification, and sequencing; (D) quantitation and analysis of 16S rRNA gene amplicons; (E) extraction and digestion of proteins from MFC anode biofilms; (F) protein identification, label-free quantification and statistical analysis; (G) metabolic pathways analysis with gene ontology and KEGG: and (H) comparison of phylogenies from DNA sequencing and metaproteomics. [file 13068_2018_1111_MOESM1_ESM.pdf]

Chignell et al., Meta-proteomics identifies protein expression distinctive to electricity-generating biofilm communities in air-cathode microbial fuel cells

Additional file 1: Supplementary Method Descriptions

A. Construction and Operation of Air-Cathode MFCs

In a single-chamber, air-cathode MFC, electrogenic microbes living in a biofilm on the surface of a conductive anode utilize that anode as an electron acceptor during anaerobic respiration. Electrons deposited to the anode flow through a circuit to the surface of a cathode, the catalytic surface of which faces the interior of the single MFC chamber. At the cathode surface, oxygen from air diffusing passively through the cathode to the interior, liquid-facing cathode surface is reduced to water. The electrochemical potential of this reduction reaction drives the flow of electrons through the MFC circuit, generating a current [Liu and Logan 2004]. Single-chamber, air-cathode MFCs were constructed based on a previous design from Liu and Logan (2004), with some modifications. Polypropylene schedule 40 pipe (IPEX, Pineville, NC), cut into 3.8 cm slices, was used for the MFC body. The liquid volume of each MFC was ~30 ml, and the circular openings were 7.0 cm^2. Non-wet-proofed carbon cloth and 30% wet-proofed carbon cloth (Fuel Cell Earth, Woburn, MA) were used for the anodes and cathodes, respectively. A mixture of polytetrafluoroethylene (PTFE) (Sigma-Aldrich, St. Louis, MO) and Vulcan XC72 carbon black powder (Cabot, Boston, MA) comprising 15 µl of 30 wt% PTFE per mg carbon powder was applied to the cathode prior to drying at 375 °C for 1 h. Diffusion layers of 60 wt% PTFE solution were applied by coating the outer, air-exposed cathode surface and then drying at 375 °C for 30 min. This process was repeated four times. Platinum catalyst applied to the interior, solution-facing side of the cathode sheet by mixing 7 µl Nafion binder and 3 µl isopropanol per gram of 10 wt% platinum on carbon (all materials from Sigma-Aldrich, St. Louis, MO) to result in 0.5 mg Pt/cm^2 cathode surface. Cathodes were allowed to dry overnight before use. Sheets of Lexan plastic 1 mm thick were cut into 10.2 cm x 10.2 cm squares, into one of which was cut a 7.0 cm^2 hole to allow for diffusion of air to the solution side of the cathode. Plumbing gasket material (Plumbcraft, Bedford Heights, OH) was placed between the Lexan squares and the anode or cathode. Titanium wire (0.012", Wytech, Rahway, NJ) pressed between the anode and MFC body as a conductive lead. All layers of the MFCs were cinched together with four screws and washers at the corners of the reactor. Nine MFCs were constructed using cathodes from the same batch.

All MFC medium components were obtained from Fisher (Pittsburgh, PA). MFC medium consisted of 5.84 g/l NaH_2PO4, 15.47 g/l Na_2HPO_4•$7H_2O$, 0.31 g/l NH_4Cl, 0.13 g/l KCl, 10 ml/l ATCC trace mineral supplement solution, 10 ml/l ATCC trace vitamin solution, and 4.08 g/l (30 mM) CH_3COONa. MFC medium was adjusted to pH 7.0 with 10 N NaOH. MFC leads were attached to a 1 kΩ external resistor (Elenco, Wheeling, IL). Voltage was recorded automatically across all MFCs every 5 min with a 16-channel Picolog 1216 multimeter (Pico Technologies, Cambridgeshire, UK) connected to a personal computer.

Inoculum for new MFCs consisted of effluent from a mature MFC originally inoculated with anaerobic digester sludge from Drake Water Reclamation Facility (Fort Collins, CO). This effluent was collected over the course of two years' operation of MFCs on 30 mM sodium acetate. Approximately once per week, 1 g/l each of ferric citrate and sodium acetate was added to this stored inoculum to enrich for metal-respiring anaerobic species. To inoculate new MFCs, 10 ml each of this stored inoculum, fresh MFC effluent, and fresh MFC medium were

added to a MFC reactor. During the biofilm enrichment process, no new inoculum was added, but 10 ml of MFC medium was replaced weekly with fresh medium.

B. Harvest of MFC Anodes

Bulk solution samples (i) were harvested 24 h after initial inoculation by transferring all bulk solution into two 15-ml conical tubes, centrifuging (3 000 x g, 20 min), discarding the supernatant, immediately freezing the pellets in liquid nitrogen, and storing the frozen pellets at -80 °C. Early anodes (ii) were harvested once appreciable but low current density (~0.05 A/m^2) was observed (~130 h). Intermediate anodes (iii) were harvested at the point of maximum current density (~0.6 A/m^2) during the batch following the first full replacement of medium (~450-500 h). That medium replacement occurred after current densities had increased appreciably above those observed for early anodes. For mature anodes (iv), anodes were harvested at the point of maximum current density (~0.8 A/m^2) after replacement of medium. Mature MFCs were operated for over two years in batch mode, replacing medium completely after current density dropped below 0.01 A/m^2.

At the harvest point, anodes were removed from MFCs and immersed briefly in sterile phosphate buffer. A small section was cut out from the center of the anode using a sterile razor blade and prepared for scanning electron microscopy. Anodes then were cut in half from top to bottom, placed in DNAse-free 50-ml conical tubes (Corning, NY), immediately flash-frozen in liquid N_2, and stored at -80 °C until extraction of proteins and DNA.

C. DNA Extraction, Amplification, and Sequencing

One anode half was thawed gradually by transferring tubes to -20 °C for 4 h and then to 4 °C for 2 h. The entire biofilm was scraped from the anode surface with a sterile razor blade into an extraction tube from the Powersoil DNA Isolation Kit (MoBio Laboratories, Inc., Carlsbad, CA, USA). The scraped anode cloth was added to the tube as well, and the tube was subjected to one freeze-thaw cycle at -80 °C to crack the biofilm. DNA extraction proceeded according to the Powersoil manufacturer's instructions.

DNA samples were amplified for sequencing by Research and Testing Laboratories (Lubbock, TX) according to their standard protocols, using a forward and reverse fusion primer. The forward primer was constructed with (5'-3') the Illumina i5 adapter (AATGATACGGCGACCACCGAGATCTACAC), an 8-10 bp barcode, a primer pad, and the 28F primer (GAGTTTGATCNTGGCTCAG). The reverse fusion primer was constructed with (5'-3') the Illumina i7 adapter (CAAGCAGAAGACGGCATACGAGAT), an 8-10 bp barcode, a primer pad, and the 388R primer (TGCTGCCTCCCGTAGGAGT). Primer pads were designed to ensure the primer pad/primer combination had a melting temperature of 63 °C-66 °C according to methods developed by the lab of Patrick Schloss (http://www.mothur.org/w/images/0/0c/Wet-lab_MiSeq_SOP.pdf). Amplifications were performed in 25 µl reactions with Qiagen HotStar Taq master mix (Qiagen Inc, Valencia, California), 1 ul of each 5uM primer, and 1 ul of template. Reactions were performed on ABI Veriti thermocyclers (Applied Biosytems, Carlsbad, California) under the following thermal profile: 95 °C for 5 min, then 35 cycles of 94 °C for 30 s, 54 °C for 40 s, 72 °C for 1 min, followed by one cycle of 72 °C for 10 min and 4 °C hold. Amplification products were visualized with eGels (Life Technologies, Grand Island, New York). Products were then pooled equimolar and each pool was size selected in two rounds using

Agencourt AMPure XP (BeckmanCoulter, Indianapolis, Indiana) in a 0.7 ratio for both rounds. Size selected pools were quantified using the Qubit 2.0 fluorometer (Life Technologies) and loaded on an Illumina MiSeq (Illumina, Inc. San Diego, California) 2x300 flow cell at 10pM.

D. Quantitation and Analysis of 16S rRNA Gene Amplicons

Results files from 16S rRNA gene sequencing by MiSeq containing counts of operational taxonomical units (OTUs) in each biological replicate sample were used to calculate the proportion of counts (i.e., relative abundance) of each taxon in each anode biofilm. Diversity of early and intermediate MFC communities was compared with two-tailed Welch's t-tests between Simpson's index values.

Multivariate analysis of MFC OTUs was conducted in R using the *vegan* package. Tests for significant differences in OTU relative abundance between communities at all developmental stages was conducted with a non-parametric multivariate analysis of variance (npMANOVA) test between all biological replicates of all developmental stages, using the *adonis* function [Buttigieg and Ramette 2014]. Pairwise Pearson's r correlations were conducted as a post-hoc analysis between each binary set of MFC developmental stages, in order to determine the developmental stage comparisons that were the driving sources of variation detected in the npMANOVA test. Additionally, a non-metric multidimensioinal scaling (NMDS) plot was constructed by generating a dissimilarity matrix with the Gower distance measure [Kuczynski et al. 2011] from a sample-by-species matrix of OTU counts by the metaMDS function in the R *vegan* package. The NMDS plot then was constructed using the ordiplot, orditorp, and ordihull functions. Since for both of these post-hoc analyses the early and mature communities showed the greatest degree of difference, a similarity percentage (SIMPER) analysis was conducted between the early and mature sample sets using the *simper* function in the *vegan* package. This analysis identifies the OTUs that contributed most to the overall differences between the communities as a whole [Clarke 1993]. Post-hoc comparisons of pairwise Pearsons correlations between each biofilm sample were conducted in R using the cor() function and plotted with ggplot(). Pearson's r coefficients were compared between sample types with a Tukey's HSD test.

E. Extraction and Digestion of Proteins from MFC Anode Biofilms

The remaining anode half was thawed gradually by transferring tubes to -20 °C for 4 h and then to 4 °C for 2 h. The entire biofilm was scraped from each anode half using a sterile razor blade. The anode half and its scraped material was placed into sterile tubes (Corning, NY), to which was added hot (95 °C) lysis buffer (50 mM ammonium bicarbonate, 1% sodium deoxycholate (SDC), pH 8.2). The samples were subjected to a round of biofilm cracking by freezing in liquid nitrogen and then thawing on ice. Samples then were sonicated on ice (50% duty cycle) for 5 min, 1 s on, 2 s off. Samples were centrifuged (5 000 x g, 10 min) and the supernatant was isolated to a low-bind, siliconized tube, which was centrifuged (14 000 x g for 20 min) to remove remaining cell debris and isolate protein extract in the supernatant.

Proteins were precipitated from the raw extract by adding an ice-cold mixture of trichloroacetic acid (TCA) in acetone (final volumetric ratio of 1:1:8 extract:TCA:acetone) and precipitating overnight in 50 ml conical tubes at -20 °C. The mixtures were centrifuged (10 000 x g, 60 min) and the supernatant was removed. Protein pellets were washed with 2 ml ice-cold acetone,

centrifuged again, acetone was removed by evaporation. Protein pellets then were resuspended in 50 mM ammonium bicarbonate, 1% sodium deoxycholate (SDC), 5% acetonitrile (ACN), pH 8.2 and sonicated for 20 s on ice (1 s on, 2 s off) to resuspend the pellet. Concentration of resuspended proteins was quantified by bicinchoninic acid (BCA) assay (Pierce Life Technologies, Carlsbad, CA). Then 50 µg of protein were combined with a volume of 100 mM dithiothreitol (DTT) to result in a final concentration of 20 mM DTT. The mixture was incubated first at 95 °C for 2 min, then at 65 °C for 30 min, to denature and reduce protein sulfide bonds. Five microliters of 375 mM iodoacetamide were added to each sample for cysteine alkylation at room temperature in the dark for 30 min. Then, a sufficient volume of 50 mM ammonium bicarbonate (pH 8.2) was added to result in a final reaction volume of 150 µl, such that the concentration of SDC was below 0.1% during the trypsin digestion. To that mixture 0.5 µl of 50 mM $CaCl_2$ was added, along with 2.5 µg (1:20 trypsin: protein) of Promega Gold mass spectrometry grade trypsin (Promega, Madison, WI). ACN was added to a final concentration of 8% (v/v) ACN. The digestion was conducted at 38 °C for 9 h, after which double digestion with one µg trypsin was conducted for 4 h. Digestion reactions were stopped by adding 5 µl 100% formic acid to decrease pH to ~2. Digestions were centrifuged (13 000 x g, 20 min) to collect any acid-precipitated SDC. A volume containing 50 µg peptides was evaporated in a speed-vap and the resulting peptide pellets were resuspended in 45 µl of 5% ACN, 0.1% formic acid. Residual detergent and contaminants were removed from 30 ul of the resuspended peptides with a C-18 spin column (Pierce Life Technologies, Carlsbad, CA), following the manufacturer's instructions. Eluted peptides were evaporated and resuspended in 2.5% acetonitrile, 0.1% formic acid for LC-MS/MS analysis (described in main text).

F. Protein Identification, Label-Free Quantification and Statistical Analysis

Results files from LC-MS/MS analysis (.wiff files from Analyst v.1.5 TR) were processed with ProteinPilot (v.4.5 beta), using a .fasta database consisting of the entire bacterial proteome (proteome filter "taxonomy: Bacteria [2]," downloaded from Uniprot 2-15-15) along with common contaminants. All three .wiff files corresponding to each technical replicate LC-MS/MS shot that was run for each biological replicate MFC anode were processed simultaneously in the database search. The search was conducted in ProteinPilot using rapid search ID and no biological modifications. False discovery rate (FDR) analysis was conducted using a decoy database consisting of reversed sequences from the bacterial database, with FDR protein identification significance threshold ≤ 0.01. An attempt was made to extract precursor ion intensities using a quantitation microapp (v. 1.0) in PeakView software (v. 1.1.1, ABsciex). Due to the large file size, however, this quantitation procedure failed, indicating the requirement for an alternative quantitation method. Peptide summaries were exported as .txt files from the .group files produced by ProteinPilot during the database search. These .txt files were imported into R statistical software (v. 3.1.2) for quantitation by spectral counting, using in-house R-scripts, as described below. First, for each biological replicate, technical replicates were separated out based on spectrum ID numbers. Since in the .txt file a given LC-MS/MS spectrum is assigned to more than one peptide, it was necessary to devise a method to handle multiple peptide-spectrum matches (PSMs) to prevent multiple counting of the same spectral evidence. ProteinPilot assigns spectra to peptides with an "Unused Score" defined as the amount of spectral evidence explained by that PSM that is not explained by a higher-ranking peptide. Only the PSM with the highest Unused Score was kept for the purpose of spectral count quantitation. This approach is similar to a "distributed" NSAF approach [Zhang et al. 2010], except that a PSM is retained based on ProteinPilot Unused Score, rather than based on the number of total PSMs assigned to that peptide. The result was a list of unique PSMs, each

with the highest observed Unused Score. Peptides then were filtered to retain only those peptides corresponding to proteins with an “N” value equal to or greater than the number of proteins reported by the ProteinPilot FDR analysis that met the 1.0% FDR cutoff. Applying this FDR cutoff, nearly all of the peptide-protein multiple assignments were to proteins from organisms of the same genus. Since downstream analysis focused on higher taxonomical categories (Genus and higher), only the top identification (also used by ProteinPilot for the protein Unused Score assignment) was retained. For cases in which a peptide was matched to homologous proteins from different genera, the identification corresponding to a genus already represented in the dataset by unique protein identifications was retained. Finally, in the few cases in which a peptide was matched to homologous proteins from different genera that were not otherwise represented in the dataset, only the first assignment provided by ProteinPilot (i.e., with the highest Unused Score) was retained.

Only proteins identified in at least one technical replicate of at least two biological replicate anodes for a condition (early or intermediate) were retained for statistical analysis. Thus, in addition to the qualifications of having a high Unused Score and meeting the 1.0% FDR cutoff as described above, proteins also had to be found across at least two biological replicate anodes to be considered for further analysis. Spectral count (SpC) was calculated for the 853 remaining proteins using the “table” function in R. Then the normalized spectral abundance factor (NSAF) was calculated for each protein in each technical replicate as previously described [Zhang et al. 2010]. The resulting value for each protein was called the “unNSAF” to reflect the fact that PSMs had been filtered according to Unused Score. For each protein, the mean unNSAF value across technical replicates for a biological replicate anode was then normalized to the count of operational taxonomical units (OTUs) of the genus corresponding to the protein, for that biological replicate anode. This normalization step was included to account for changes in the relative abundance of that genus in the consortium when comparing protein expression between developmental stages. Then $\log_2$(unNSAF) was calculated for each protein in each biological replicate MFC. For those proteins found in both early and intermediate anode biofilms, the resulting value was used to compute fold-change ratios between early and intermediate developmental stages as well as to conduct a two-tailed, homoscedastic Student’s t-test between the two developmental stages. Since a multiple testing correction [Storey et al. 2002] resulted in no proteins with $q<0.05$ despite the clear left peak in the p-value histogram (SI Figure S8), only fold-change and p-value cutoffs were used as criteria forproteins of interest (POIs) , as suggested recently [Pascovici et al. 2016]. Those cutoffs were $\log_2$(intermediate/early) >1 or -1 and $p<0.05$. For proteins identified in only one of the two MFC conditions, significance testing was conducted at the pathway level, as described below.

G. Metabolic Pathways Analysis with Gene Ontology and KEGG

The set of intermediate MFC proteins was compared with the set of early MFC proteins, using a Fisher’s Exact Test based on the FatiGO algorithm [Al-Shahrour et al. 2004] implemented in Blast2GO (v.3.1.0, www.blast2go.com). A Benjamini-Hochberg FDR multiple testing correction (FDR < 0.05) [Benjamini and Hochberg 1995] was applied in the test to identify Gene Ontology pathways that were significantly enriched among the proteins from each developmental stage. This type of significance testing seems especially appropriate for samples containing different genera, since comparable proteins from different genera contribute to the determination of significance of entire pathways, rather than individual proteins.

For analysis with the KEGG database (http://www.genome.jp/kegg/) the combined set of proteins that were either a UDPI or a POI more abundant in the intermediate anode biofilm

were assigned to KEGG pathways using the KEGG GhostKOALA tool (http://www.kegg.jp/ghostkoala/). This tool, appropriate for use with metaproteomics datasets, bins submitted proteins into KEGG functional modules and summarizes protein pathway and taxonomic distributions.

H. Comparison of Phylogenies from DNA Sequencing and Metaproteomics

For each MFC biofilm, species composition was quantitated both in terms of relative abundance of MiSeq OTUs and in terms of relative abundance of proteins associated with genera by the KEGG GhostKOALA tool. These two methods generally agreed on broad phylogenetic trends. As reported in the main text, both MiSeq and GhostKOALA methods showed greater diversity in the intermediate biofilms than in the early biofilms. The linear regression model of all relative abundance values showed a strong overall correlation (R^2 = 0.914, p-value < $2.2e^{-16}$) between the GhostKOALA and MiSeq datasets. Eight of the 10 greatest residuals in the linear model were observed for non-*Enterobacter Gammaproteobacteria*, *Betaproteobacteria* or *Deltaproteobacteria* in the intermediate MFCs. For seven of those residuals, the relative abundance of OTUs was less than the relative abundance of proteins assigned to that taxon (i.e., residuals were greater than 0), perhaps due to the greater number of total taxa identified by the MiSeq method (SI Figure S7).

The two methods for determining phylogeny—OTUs and proteins—generally agreed on the identities of the most abundant taxa. For both methods, non-*Enterobacter Gammaproteobacteria* had by far the greatest relative abundance in the early MFCs (83.2 ± 4.1% of OTUs and 74.5 ± 2.9% of GhostKOALA protein identifications). Further, the top five most abundant taxa were the same for MiSeq and GhostKOALA methods, except for *Deltaproteobacteria*, the taxon containing *Geobacter*. That taxon had the third-highest relative abundance in early MFCs based on GhostKOALA categorization of proteins, while it was the eighth most abundant class based on OTUs. For intermediate biofilms, the top five most abundant taxa were the same for both classification methods, though with some differences in rank (SI Table S5). As with the early biofilms, the differences in rank of taxa between the two methods was most pronounced for *Deltaproteobacteria*. This class was the second most abundant in intermediate MFCs according to GhostKOALA classification of proteins (22.8 ± 3.6% of identified proteins) but fourth most abundant according to OTUs (11.3 ± 4.2% of OTUs). Both methods indicated that *Gammaproteobacteria* remained the most abundant taxon in intermediate MFCs (27.9 ± 5.0% of proteins and 25.4 ± 4.3% of OTUs). Several previous metaproteomics studies compared phylogenies gleaned from 16S rRNA gene sequences with those reflected in protein identifications [Qu et al. 2012]. This type of comparison serves as a useful validation of a portion of the proteomics dataset, as well as an indication of the community coverage achieved by protein extraction and identification.

WORKS CITED

Al-Shahrour F, Diaz-Uriarte R, Dopazo J. FATIGO: a web tool for finding significant associations of Gene Ontology terms with groups of genes. Bioinformatics 2010;20:578-580.

Benjamini Y and Hochberg Y. Controlling the false discovery rate: a powerful and practical powerful approach to multiple testing. J Royal Stat. Soc, Series B. 1995;57:289-300.

Additional file 1

Buttigieg PL and Ramette A. A guide to statistical analysis in microbial ecology: a community-focused, living review of multivariate data analyses. FEMS Microb. Ecol. 2014;90: 534-550.

Clarke, KR. Non-parametric multivariate analyses of changes in community structure. Austral. Ecol. 1993;18:117-143.

Kuczynski J, Liu Z, Lozupone C, McDonald D, Fierer N, Knight R. Microbial community resemblance methods differ in their ability to detect biologically relevant patterns. Nat. Methods 2010;7:813-819.

Liu H and Logan BE. Electricity generation using an air-cathode single chamber microbial fuel cell in the presence and absence of a proton exchange membrane. Environ. Sci. Tech. 2004;38:4040-4046.

Pascovici D, Handler DCL, Wu JX, Haynes PA. Multiple testing corrections in quantitative proteomics: a useful but blunt tool. Proteomics 2016;16:2448-2453

Qu Y, Feng Y, Wang X, Logan BE. Use of a coculture to enable current production by *Geobacter sulfurreducens*. Appl. Env. Microbiol. 2012;78:3484-3487

Storey JD. A direct approach to false discovery rates. Stat. Methodol. Ser. B. 2002;64:479-498.

Zhang Y, Wen Z, Washburn MP, Florens L. Refinements to label free proteome quantitation: how to deal with peptides shared by multiple proteins. Anal. Chem. 2010;82:2272-2281.
