# Supplementary material for: Meta-proteomic analysis of protein expression distinctive to electricity-generating biofilm communities in air-cathode microbial fuel cells
Source: Biotechnol Biofuels. 2018 Apr 23;11:121. doi: 10.1186/s13068-018-1111-2 (PMC5913794; doi:10.1186/s13068-018-1111-2)
Supplement: Supplementary file 2 — Additional file 2: Figure S1. Scanning electron microscope images (400× or 3,000× magnification) of MFC anode carbon cloth fibers at (A) non-inoculated, (B) early, (C) intermediate, and (C) mature stages of biofilm development. Figure S2. Current density for replicates of early (top) and intermediate (bottom) MFCs (inoculum originally derived from anaerobic digester sludge) after enrichment on batches of 30 mM acetate. Figure S3. Current density of a mature MFC (inoculum originally derived from anaerobic digester sludge) after enrichment for over 2 years on batches of 30 mM acetate. Figure S4. Non-metric multidimensional scaling (NMDS) plot of MFC anode OTUs along two principal coordinates. OTUs were identified by MiSeq sequencing of MFC anode 16S rRNA gene amplicons from three biological replicates of four different developmental conditions: S (solution), E (early biofilm), I (intermediate biofilm), M (mature biofilm). In the R vegan package, a Gower dissimilarity matrix was constructed with the metaMDS function from a sample-by-species matrix of OTU counts. The ordiplot, orditorp, and ordihull functions then were used to generate the NMDS plot. Similarity in relative abundance of genera is represented as spatial proximity within the two principal coordinates. Samples for S and E were clustered close enough together to obscure distinction of sample replicates. Figure S5. Linear regression of OTUs from MiSeq sequencing of 16S rRNA gene amplicons (“otu”) against proteins identified by GhostKOALA (“koala”) with respect to relative abundance of taxa. The linear regression was performed in R using the lm function on paired relative abundance values for each taxon in each biological replicate anode biofilm, excluding the intercept. Adjusted R2 = 0.914, p-value < 2.2e−16. Figure S6. Residuals vs. fitted plots for linear regression of taxon relative abundances, as quantified by GhostKOALA identification of proteins (“koala”) and OTUs from MiSeq sequencing of 16S rRNA gene [file 13068_2018_1111_MOESM2_ESM.pdf]

Chignell et al., Meta-proteomics identifies protein expression distinctive to electricity-generating biofilm communities in air-cathode microbial fuel cells

Additional file 2: Supplementary Figures S1-S8

Figure S1: Scanning electron microscope images (400X or 3 000X magnification) of MFC anode carbon cloth fibers at (A) non-inoculated, (B) early, (C) intermediate, and (C) mature stages of biofilm development.

Figure S2: Current density for replicates of early (top) and intermediate (bottom) MFCs (inoculum originally derived from anaerobic digester sludge) after enrichment on batches of 30 mM acetate.

Figure S3: Current density of a mature MFC (inoculum originally derived from anaerobic digester sludge) after enrichment for over two years on batches of 30 mM acetate.

Figure S4: Non-metric multidimensional scaling (NMDS) plot of MFC anode OTUs along two principal coordinates. OTUs were identified by MiSeq sequencing of MFC anode 16S rRNA gene amplicons from three biological replicates of four different developmental conditions: S (solution), E (early biofilm), I (intermediate biofilm), M (mature biofilm). In the R *vegan* package, a Gower dissimilarity matrix was constructed with the metaMDS function from a sample-by-species matrix of OTU counts. The ordiplot, orditorp, and ordihull functions then were used to generate the NMDS plot. Similarity in relative abundance of genera is represented as spatial proximity within the two principal coordinates. Samples for S and E were clustered close enough together to obscure distinction of sample replicates.

Figure S5: Linear regression of OTUs from MiSeq sequencing of 16S rRNA gene amplicons ("otu") against proteins identified by GhostKOALA ("koala") with respect to relative abundance of taxa. The linear regression was performed in R using the *lm* function on paired relative abundance values for each taxon in each biological replicate anode biofilm, excluding the intercept. Adjusted $R^2 = 0.914$, p-value < $2.2e^{-16}$.

Figure S6: Residuals vs. fitted plots for linear regression of taxon relative abundances, as quantified by GhostKOALA identification of proteins ("koala") and OTUs from MiSeq sequencing of 16S rRNA gene amplicons ("otu"). The ten pairwise comparisons with the greatest positive or negative residuals are labeled.

Figure S7: Venn diagrams of numbers of protein identifications identified in and shared between three biological replicates of (A) early MFC anodes and (B) intermediate MFC anodes.

Figure S8: Venn diagram of proteins identified in early and intermediate anode development stages. Proteins in the overlapping area were identified in both conditions in at least one technical replicate of at least two biological replicates. The upper section of the overlapping area represents DEPs significantly more abundant in the intermediate condition, while the lower section represents DEPs significantly more abundant in the early condition.

Additional file 2

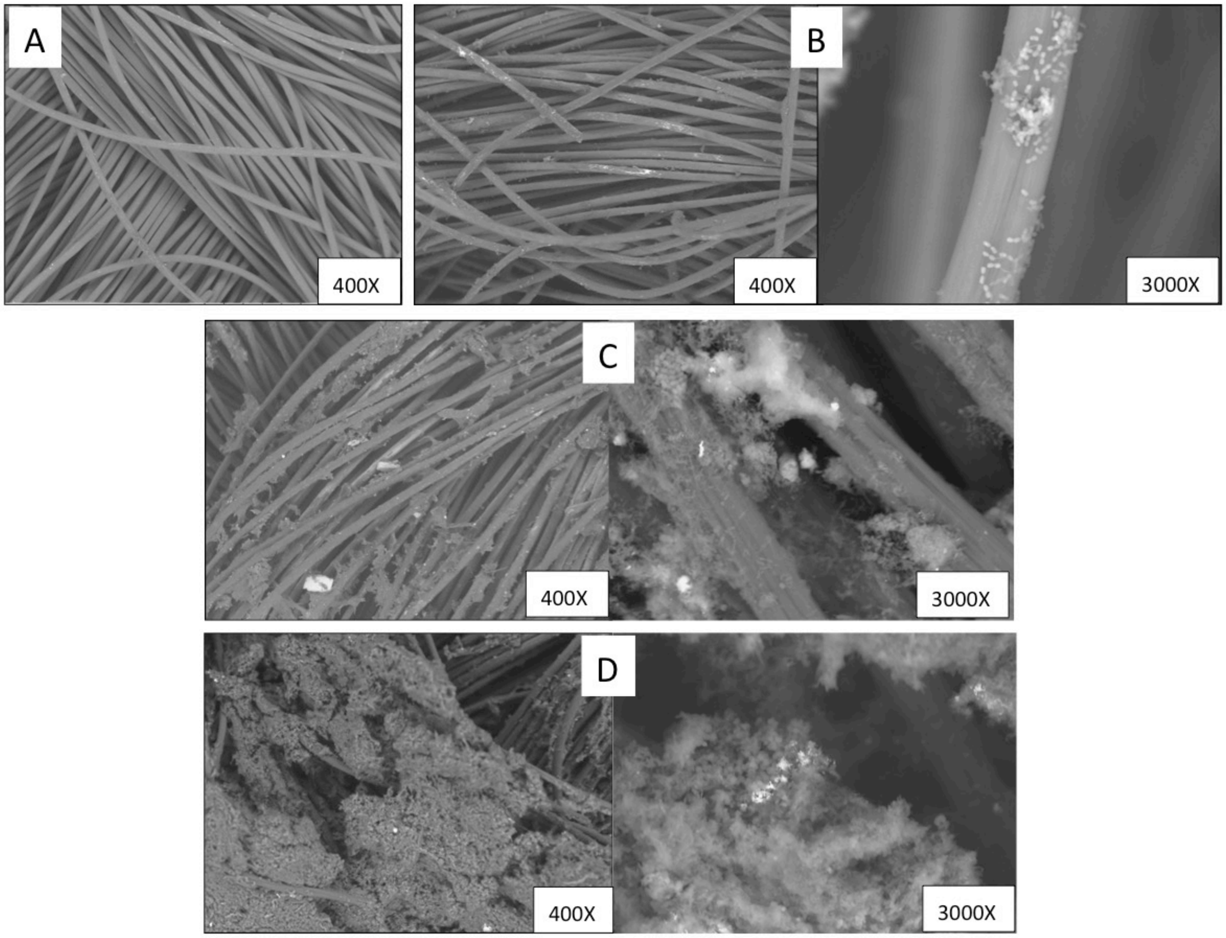

Figure S1: Scanning electron microscope images (400X or 3 000X magnification) of MFC anode carbon cloth fibers at (A) non-inoculated, (B) early, (C) intermediate, and (C) mature stages of biofilm development.

Additional file 2

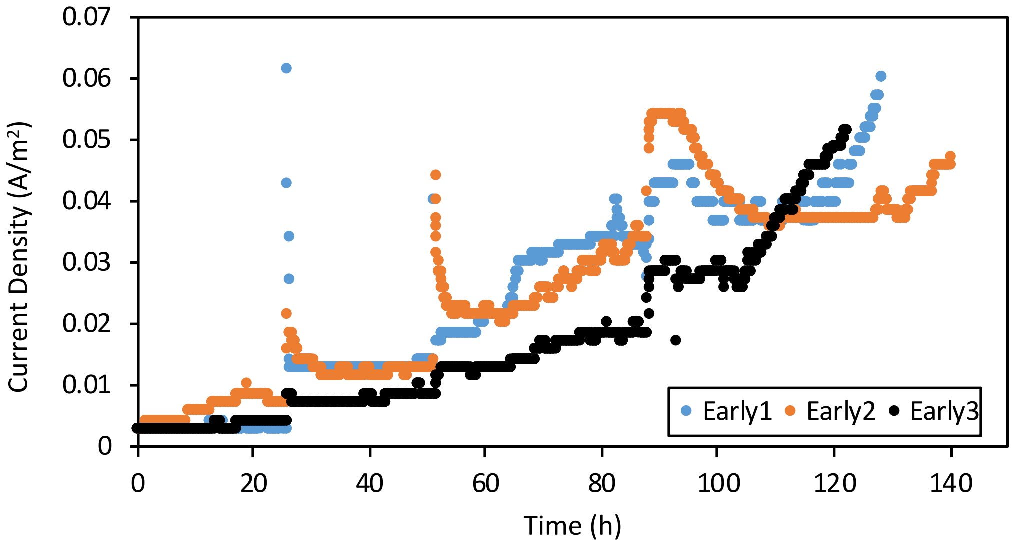

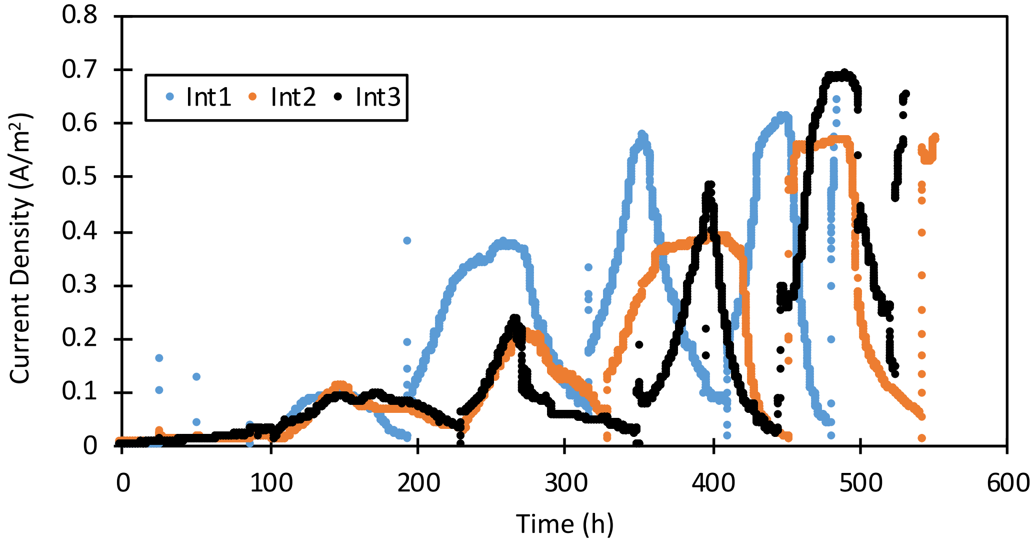

Figure S2: Current density for replicates of early (top) and intermediate (bottom) MFCs (inoculum originally derived from anaerobic digester sludge) after enrichment on batches of 30 mM acetate.

Additional file 2

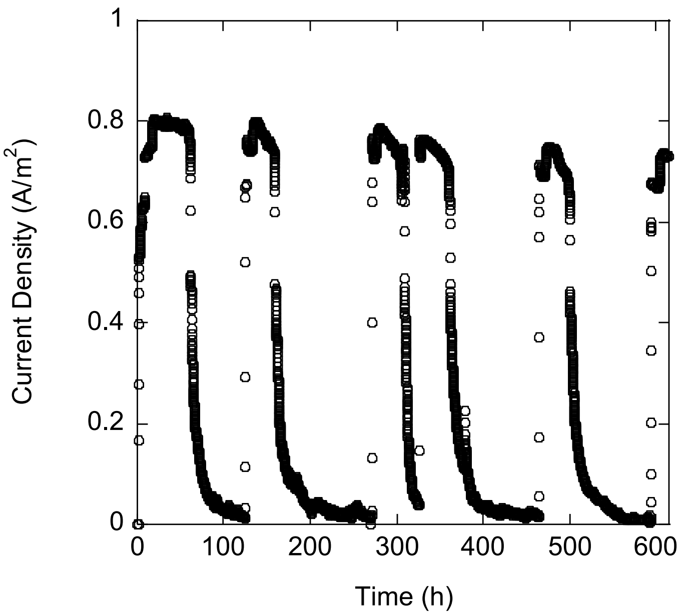

Figure S3: Current density of a mature MFC (inoculum originally derived from anaerobic digester sludge) after enrichment for over two years on batches of 30 mM acetate.

Additional file 2

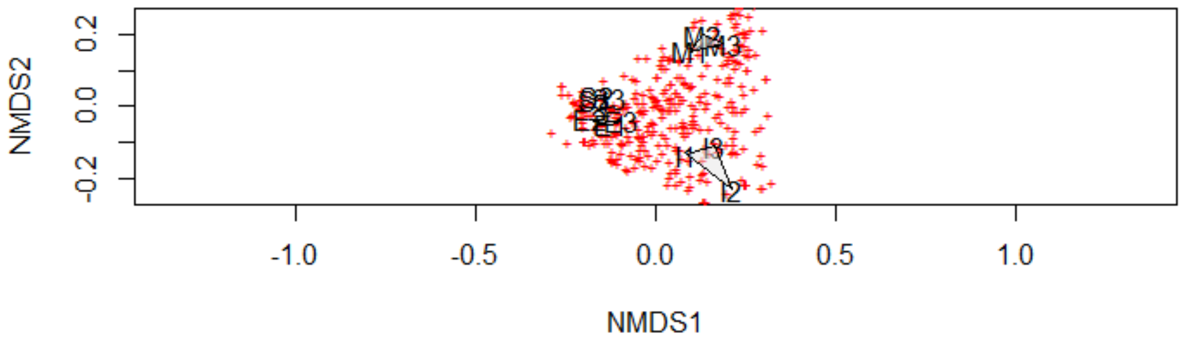

Figure S4: Non-metric multidimensional scaling (NMDS) plot of MFC anode OTUs along two principal coordinates. OTUs were identified by MiSeq sequencing of MFC anode 16S rRNA gene amplicons from three biological replicates of four different developmental conditions: S (solution), E (early biofilm), I (intermediate biofilm), M (mature biofilm). In the R *vegan* package, a Gower dissimilarity matrix was constructed with the metaMDS function from a sample-by-species matrix of OTU counts. The ordiplot, orditorp, and ordihull functions then were used to generate the NMDS plot. Similarity in relative abundance of genera is represented as spatial proximity within the two principal coordinates. Samples for S and E were clustered close enough together to obscure distinction of sample replicates.

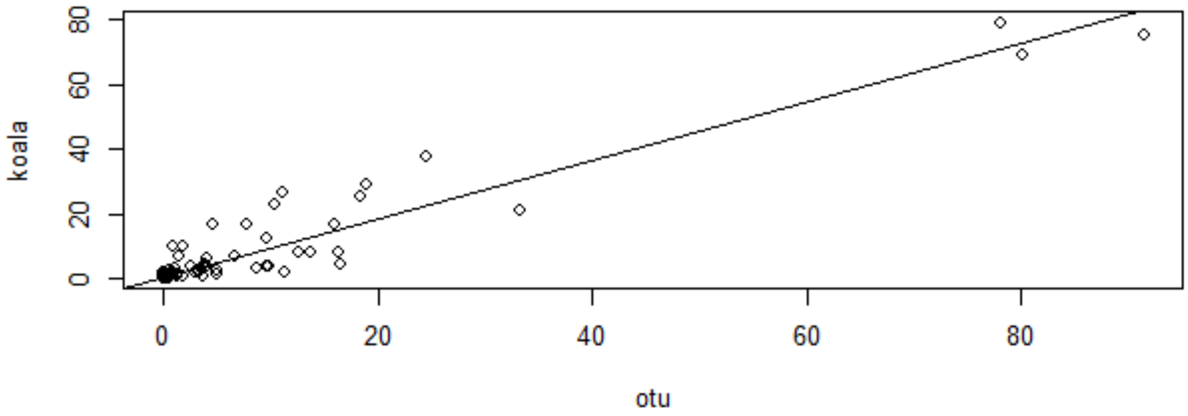

Figure S5: Linear regression of OTUs from MiSeq sequencing of 16S rRNA gene amplicons ("otu") against proteins identified by GhostKOALA ("koala") with respect to relative abundance of taxa. The linear regression was performed in R using the *lm* function on paired relative abundance values for each taxon in each biological replicate anode biofilm, excluding the intercept. Adjusted $R^2 = 0.914$, p-value $< 2.2e^{-16}$.

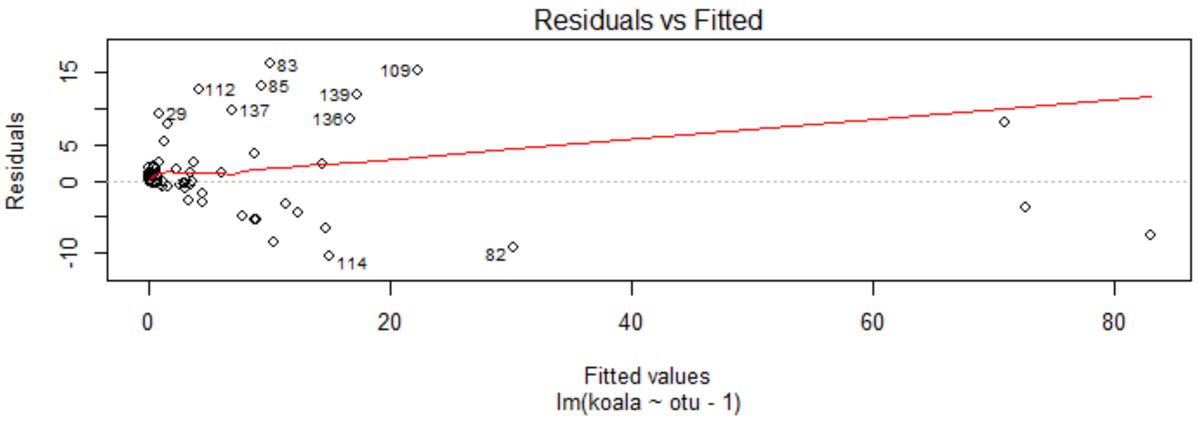

Figure S6: Residuals vs. fitted plots for linear regression of taxon relative abundances, as quantified by GhostKOALA identification of proteins ("koala") and OTUs from MiSeq sequencing of 16S rRNA gene amplicons ("otu"). The ten pairwise comparisons with the greatest positive or negative residuals are labeled.

Additional file 2

(A)

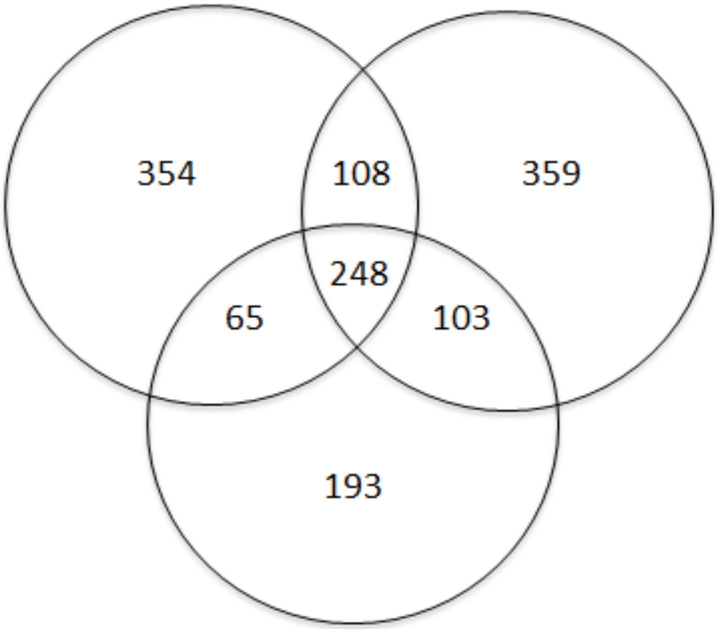

(B)

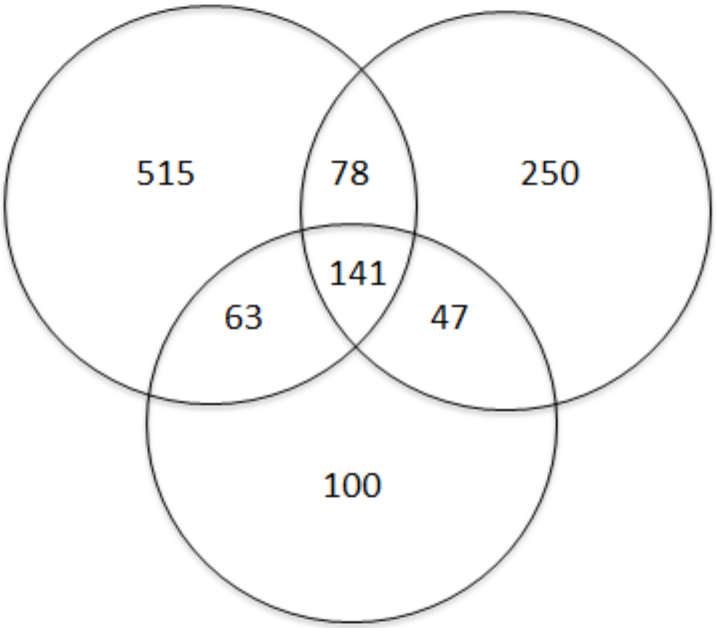

Figure S7: Venn diagrams of numbers of protein identifications identified in and shared between three biological replicates of (A) early MFC anodes and (B) intermediate MFC anodes.

Additional file 2

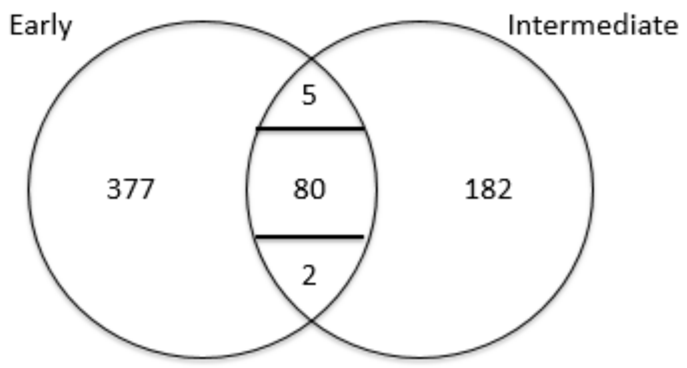

Figure S8: Venn diagram of proteins identified in early and intermediate anode development stages. Proteins in the overlapping area were identified in both conditions in at least one technical replicate of at least two biological replicates. The upper section of the overlapping area represents DEPs significantly more abundant in the intermediate condition, while the lower section represents DEPs significantly more abundant in the early condition.
