# Supplementary material for: Meta-proteomic analysis of protein expression distinctive to electricity-generating biofilm communities in air-cathode microbial fuel cells
Source: Biotechnol Biofuels. 2018 Apr 23;11:121. doi: 10.1186/s13068-018-1111-2 (PMC5913794; doi:10.1186/s13068-018-1111-2)
Supplement: Supplementary file 3 — Additional file 3: Table S1. Comparison of OTU relative abundance between four MFC developmental conditions: early (E), intermediate (I), mature (M) and solution (S), as identified by MiSeq sequencing of 16S rRNA genes. Each developmental stage was represented by three independent biological replicate samples. A nonparametric MANOVA p-value was generated by a comparison between all conditions. As a post hoc test between conditions, each replicate sample was compared against each replicate sample in the other condition by Pearson’s pairwise correlation, thereby generating mean and standard deviation Pearsons’ r coefficients for each pairwise comparison. Coefficients with different subscript letters indicate Pearson’s r coefficients that differ significantly (Tukey’s HSD test, p < 0.05). Table S2. Relative abundance across developmental stages of OTUs contained within different classes of the phylum Proteobacteria. Error terms represent ± one standard deviation across three separate MFC anode (or solution) samples. Table S3. Relative abundance across developmental stages of the three genera responsible for the majority of significant differences between early and mature MFC anode communities. Error terms represent ± one standard deviation across three separate MFC anode (or solution) samples. Table S4. Prominent OTUs (relative abundance greater than or equal to 1.0%) in MFC communities at each stage of development. The OTU column shows the identification number for each particular OTU. Solution samples were from the bulk solution of the MFCs 24 h after inoculation. The column for %OTUs signifies the percentage of OTUs in that sample that were identified as the specified OTU. The error term represents ± one standard deviation (SD) across three biological replicate samples. Table S5. Selected UDPIs categorized by broad metabolic process, as determined by KEGG, GO, Uniprot, and literature review. Table S6. Complete relative abundance values (mean % OTUs in a sample) for [file 13068_2018_1111_MOESM3_ESM.pdf]

Chignell et al., Meta-proteomics identifies protein expression distinctive to electricity-generating biofilm communities in air-cathode microbial fuel cells

Additional File 3: Supplementary Tables S1-S6

Table S1: Comparison of OTU relative abundance between four MFC developmental conditions: early (E), intermediate (I), mature (M) and solution (S), as identified by MiSeq sequencing of 16S rRNA genes. Each developmental stage was represented by three independent biological replicate samples. A nonparametric MANOVA p-value was generated by a comparison between all conditions. As a post-hoc test between conditions, each replicate sample was compared against each replicate sample in the other condition by Pearson's pairwise correlation, thereby generating mean and standard deviation Pearsons' r coefficients for each pairwise comparison. Coefficients with different subscript letters indicate Pearson's r coefficients that differ significantly (Tukey's HSD test, $p<0.05$).

Table S2: Relative abundance across developmental stages of OTUs contained within different classes of the phylum Proteobacteria. Error terms represent ± one standard deviation across three separate MFC anode (or solution) samples.

Table S3: Relative abundance across developmental stages of the three genera responsible for the majority of significant differences between early and mature MFC anode communities. Error terms represent ± one standard deviation across three separate MFC anode (or solution) samples.

Table S4: Prominent OTUs (relative abundance greater than or equal to 1.0%) in MFC communities at each stage of development. Solution samples were from the bulk solution of the MFCs 24 h after inoculation. The column for OTU shows the identifier for specific OTUs. The column for %OTUs signifies the percentage of total OTUs in that sample that were identified as the specified OTU. The error term represents ± one standard deviation (SD) across three biological replicate samples.

Table S5: Selected UDPIs categorized by broad metabolic process, as determined by KEGG, GO, Uniprot, and literature review.

Table S6: Complete relative abundance values (mean % OTUs in a sample) for taxa in common between GhostKOALA annotation of proteins and OTUs from MiSeq sequencing of 16S rRNA gene amplicons from early and intermediate MFC anode biofilm samples. Relative abundances of OTUs are also shown for the solution and mature biofilm samples.

Additional file 3

Table S1: Comparison of OTU relative abundance between four MFC developmental conditions: early (E), intermediate (I), mature (M) and solution (S), as identified by MiSeq sequencing of 16S rRNA genes. Each developmental stage was represented by three independent biological replicate samples. A nonparametric MANOVA p-value was generated by a comparison between all conditions. As a post-hoc test between conditions, each replicate sample was compared against each replicate sample in the other condition by Pearson's pairwise correlation, thereby generating mean and standard deviation Pearsons' r coefficients for each pairwise comparison. Coefficients with different subscript letters indicate Pearson's r coefficients that differ significantly (Tukey's HSD test, $p<0.05$).

**Comparison**	**npMANOVA p-value or Pearson's r coefficient**
EIMS	0.00099
EI	0.53 ± 0.22^{a}
EM	0.01 ± 0.01^{b}
ES	0.52 ± 0.30^{a}
IM	0.40 ± 0.23^{a}
IS	0.38 ± 0.20^{a}
MS	0.005 ± 0.004^{b}

Additional file 3

Table S2: Relative abundance across developmental stages of OTUs contained within different classes of the phylum Proteobacteria. Error terms represent ± one standard deviation across three separate MFC anode (or solution) samples.

Class	Solution (%)	Early (%)	Intermediate (%)	Mature (%)
Gammaproteobacteria	90.9 ± 3.3	83.4 ± 7.1	25.6 ± 7.2	1.1 ± 0.6
Deltaproteobacteria	0.1 ± 0.1	0.5 ± 0.5	11.3 ± 7.2	70.1 ± 3.7
Alphaproteobacteria	0.3 ± 0.1	4.1 ± 0.9	14.3 ± 2.0	1.5 ± 0.9
Betaproteobacteria	3.4 ± 1.3	1.4 ± 0.4	11.6 ± 4.1	1.5 ± 0.3
Epsilonproteobacteria	1.7 ± 1.6	0.7 ± 0.2	0.0 ± 0.0	0.0 ± 0.0

Table S3: Relative abundance across developmental stages of the three genera responsible for the majority of significant differences between early and mature MFC anode communities. Error terms represent ± one standard deviation across three separate MFC anode (or solution) samples.

Genus	Class	Planktonic (%)	Early (%)	Intermediate (%)	Mature (%)
Acinetobacter	*Gammaproteobacteria*	25.8 ± 11.3	27.3 ± 28.5	6.7 ± 6.2	0.4 ± 0.2
Pseudomonas	*Gammaproteobacteria*	58.1 ± 18.9	55.1 ± 33.1	17.7 ± 9.9	0.6 ± 0.3
Geobacter	*Deltaproteobacteria*	0.1 ± 0.0	0.5 ± 0.5	11.0 ± 7.1	68.7 ± 3.6

Additional file 3

Table S4: Prominent OTUs (relative abundance greater than or equal to 1.0%) in MFC communities at each stage of development. Solution samples were from the bulk solution of the MFCs 24 h after inoculation. The column for %OTUs signifies the percentage of total OTUs in that sample that were identified as the specified OTU. The error term represents ± one standard deviation (SD) across three biological replicate samples.

OTU	Genus	% OTUs (± SD)
Planktonic (Solution)		
356	*Pseudomonas*	30.6 ± 27.6
96	*Pseudomonas*	26.1 ± 25.4
132	*Acinetobacter*	25.8 ± 11.3
274	Unknown *Enterobacteriaceae*	6.5 ± 5.2
68	*Comamonas*	2.5 ± 1.3
189	*Arcobacter*	1.7 ± 1.6
154	Unknown *Bacteria*	1.2 ± 1.2
Early		
96	*Pseudomonas*	53.3 ± 32.8
132	*Acinetobacter*	27.1 ± 28.3
78	*Flavobacterium*	4.4 ± 2.8
380	Unknown *Rhizobiales*	3.4 ± 0.8
236	*Rhodococcus*	1.0 ± 1.7
356	*Pseudomonas*	1.0 ± 0.4
Intermediate		
96	*Pseudomonas*	15.6 ± 10.0
333	*Geobacter*	11.0 ± 7.1
380	Unknown *Rhizobiales*	9.2 ± 0.8
132	*Acinetobacter*	6.5 ± 6.1
204	Unknown *Synergistales*	6.3 ± 4.1
198	*Thauera*	5.9 ± 1.9
78	*Flavobacterium*	4.4 ± 3.7
236	*Rhodococcus*	4.0 ± 3.2
391	*Clostridium*	3.3 ± 3.1
154	Unknown *Bacteria*	3.2 ± 3.6
288	*Alcaligenes*	2.1 ± 1.7
152	Unknown *Actinomycetales*	1.9 ± 2.0
356	*Pseudomonas*	1.7 ± 0.8
59	Unknown *Bacteria*	1.2 ± 0.6
165	*Gordonia*	1.2 ± 1.1
314	*Myroides*	1.2 ± 1.6
296	*Brevundimonas*	1.1 ± 0.5
Mature		
333	*Geobacter*	68.7 ± 3.6
154	Unknown *Bacteria*	13.2 ± 7.2
204	Unknown *Synergistales*	2.3 ± 0.5
30	*Actinomyces*	2.0 ± 2.7

Additional file 3

Table S5: Selected UDPIs categorized by broad metabolic process, as determined by KEGG, GO, Uniprot, and literature review.

Uniprot ID	Protein Name	Function or Pathway	Genus
	**Central Carbon Metabolism**		
A5GEB7	Citrate synthase	TCA cycle	*Alcaligenes*
U7U728	Aconitate hydratase B	TCA cycle	*Alcaligenes*
Q1JW62	Aconitate hydratase B	TCA cycle	*Desulfuromonas*
I4N0T6	Aconitate hydratase B	TCA cycle	*Pseudomonas*
U7U739	Citrate synthase	TCA cycle	*Alcaligenes*
U7U7F2	Citrate synthase	TCA cycle	*Alcaligenes*
U7U7F8	Malate dehydrogenase	TCA cycle	*Alcaligenes*
Q74D54	Isocitrate dehydrogenase	TCA cycle	*Geobacter*
Q74EG8	Fumarate hydratase, class I	TCA cycle	*Geobacter*
I7FK92	Phosphoglycerate kinase	Glycolysis/gluconeogenesis	*Geobacter*
V9WRF6	Glyceraldehyde-3-phosphate dehydrogenase	Glycolysis/gluconeogenesis	*Pseudomonas*
A0A081GJT4	Glycogen debranching protein	Glycolysis/gluconeogenesis	*Cyanobium*
W7YDT5	Enolase	Glycolysis/gluconeogenesis	*Saccharicrinis*
N6YGD2	Phosphoenolpyruvate carboxykinase	Gluconeogenesis	*Thauera*
N6XEP4	Isocitrate lyase	Glyoxylate cycle	*Thauera*
	**Anaerobic Metabolism**		
Q74D51	2-oxoglutarate:ferredoxin oxidoreductase, alpha subunit (KorA)	Anaerobic TCA cycle	*Geobacter*
Q74D50	2-oxoglutarate:ferredoxin oxidoreductase, thiamin diphosphate-binding subunit (KorB)	Anaerobic TCA cycle	*Geobacter*
Q74D49	2-oxoglutarate:ferredoxin oxidoreductase, gamma subunit (KorC)	Anaerobic TCA cycle	*Geobacter*
Q74GZ6	Pyruvate-flavodoxin oxidoreductase (Por)	Anaerobic TCA cycle	*Geobacter*
E3PT29	Pyruvate-flavodoxin oxidoreductase (Por)	Anaerobic TCA cycle	*Clostridium*

B8J4R0	Sulfite reductase, dissimilatory-type alpha subunit	Anaerobic respiration	*Desulfovibrio*
B2YHF8	Dissimilatory sulphite reductase beta subunit (Fragment)	Anaerobic respiration	*Uncultured*
Q8EKJ1	Nitrate-inducible formate dehydrogenase molybdopterin-binding subunit FdnG	Anaerobic respiration	*Shewanella*
	**Acetate Metabolism**		
Q74FU6	NADPH-Fe(3+) oxidoreductase subunit alpha (SfrA)	Acetate metabolism.	*Geobacter*
Q74FU5	NADPH-Fe(3+) oxidoreductase subunit beta (SfrB)	Acetate metabolism.	*Geobacter*
Q74GS1	Succinyl:acetate coenzyme A transferase	Acetyl-CoA synthesis	*Geobacter*
	**Fatty Acid Metabolism**		
J0JLY0	Acetyl-CoA acetyltransferase	Fatty acid biosynthesis	*Alcaligenes*
Q74BM2	Acetyl-CoA carboxylase, biotin carboxylase component (Acc-ase)	Fatty acid biosynthesis	*Geobacter*
Q74CR7	3-oxoacyl-[acyl-carrier-protein] synthase 2 (EC 2.3.1.179)	Fatty acid biosynthesis	*Geobacter*
T0AZR6	Acyl carrier protein (ACP)	Fatty acid biosynthesis	*Thauera*
S9ZEL5	3-ketoacyl-ACP reductase (EC 1.1.1.36)	Fatty acid biosynthesis	*Thauera*
A0A022LGX0	Long-chain fatty acid--CoA ligase	Fatty acid β-oxidation	*Dietzia*
S9ZIA5	Acyl-CoA dehydrogenase	Fatty acid β-oxidation	*Thauera*
Q747G7	Biotin-dependent acyl-CoA carboxylase, carboxyltransferase subunit	Fatty acid β-oxidation	*Geobacter*
Q39UX8	Short-chain acyl-CoA dehydrogenase	Fatty acid β-oxidation	*Geobacter*

Additional file 3

	**Membrane Proteins**		
U1XWB5	Membrane protein	Unknown	*Alcaligenes*
U7U8X8	Membrane protein	Unknown	*Alcaligenes*
X5HVW6	Membrane protein	Unknown	*Aeromonas*
H1RM45	Gram-negative type outer membrane porin protein	Transport	*Comamonas*
B9MDB0	Porin Gram-negative type	Transport	*Acidovorax*
X5HGU5	Porin	Transport	*Aeromonas*
A0A022LPU2	ABC transporter substrate-binding protein	Transport	*Dietzia*
A0A073ISB0	Branched-chain amino acid ABC transporter substrate-binding protein	Transport	*Synergistes*
L7X6F0	Membrane protein (Outer membrane protein A)	Receptor; transport	*Aeromonas*
Q74AK2	Sodium/solute symporter family protein	Transport	*Geobacter*
Q39Y99	Outer membrane channel, putative	Transport	*Geobacter*
Q74E95	Sodium/solute symporter family protein	Transport	*Geobacter*
K2N8B7	Basic membrane lipoprotein	Transport	*Nitratireductor*
K2PP47	Cationic amino acid ABC transporter periplasmic binding protein	Transport	*Nitratireductor*
W6XM61	ABC-type transporter, periplasmic subunit	Transport	*Burkholderia*
A0A075PCY9	Porin	Transport	*Pseudomonas*
Q74FD9	Lipoprotein cytochrome c	Electron transfer	*Geobacter*
Q74GH2	Cytochrome c,, and cytochrome b	Electron transfer	*Geobacter*
Q39RK5	ResB-like family cytochrome c	Electron transfer	*Geobacter*
Q74FJ5	ResB-like family cytochrome c biogenesis protein	Electron transfer	*Geobacter*
Q74GA2	NADH dehydrogenase I, G subunit	Electron transport chain	*Geobacter*
A0A067A3Q5	Outer membrane insertion C-terminal signal domain protein	Protein insertion	*Pseudomonas*
W8Q8M8	Outer membrane protein assembly factor BamA	Protein insertion	*Pseudomonas*
A0A077F872	Outer membrane protein H1	Antibiotic resistance	*Pseudomonas*
A0A081UU63	Maltoporin (Maltose-inducible porin)	Transport	*Aeromonas*

A0A085ETL9	Various polyols ABC transporter, periplasmic substrate-binding protein	Transport	*Devosia*
	**Stress Response or Interactions**		
Q74EI7	Phage tail sheath protein, putative	Phage	*Geobacter*
N6XZL4	Tail sheath protein	Phage	*Thauera*
Q74FS1	Cysteine synthase A	Contact-dependent inhibition	*Geobacter*
Q74E06	Superoxide dismutase	Oxidative stress	*Geobacter*
N6ZL72	Superoxide dismutase	Oxidative stress	*Thauera*
N6Y581	Phenylacetic acid degradation protein PaaD	Response to toxic aromatic	*Thauera*
U7UB80	Alkyl hydroperoxide reductase subunit C	Oxidative stress	*Alcaligenes*
Q74E46	Universal stress protein	Stress	*Geobacter*
	**Nitrogen Metabolism**		
E3PUH2	Glutamate dehydrogenase	Amino acid biosynthesis	*Clostridium*
G9PT06	Glutamate dehydrogenase	Amino acid biosynthesis	*Synergistes*
R5PD20	Glutamate dehydrogenase	Amino acid biosynthesis	*Odoribacter*
Q74DL1	Glutamate dehydrogenase	Amino acid biosynthesis	*Geobacter*
U7UGI7	Glutamate dehydrogenase	Amino acid biosynthesis	*Prevotella*
A0A022LGN0	Glutamate-binding protein	Amino acid biosynthesis	*Dietzia*
Q760A4	Nitrite reductase (Fragment)	Denitrification or nitrification	*uncultured*
U1YC31	Nitrous-oxide reductase	Denitrification	*Alcaligenes*
A1KA74	NosZ protein	Denitrification	*Azoarcus*
A7UMG8	Nitrous oxide reductase	Denitrification	*uncultured*
Q747H2	Rubredoxin:oxygen/nitric oxide oxidoreductase	Nitric oxide detoxification	*Geobacter*
Q74BM9	Nitrogen fixation protein NifU	Nitrogen fixation	*Geobacter*
E3PUA0	Hydroxylamine reductase/nitrate reductase (EC 1.7.99.1)	Nitrogen assimilation; nitric oxide production	*Clostridium*

Additional file 3

Uniprot ID	Protein Name	Function or Pathway	Genus
	**Central Carbon Metabolism**		
A5GEB7	Citrate synthase	TCA cycle	*Alcaligenes*
U7U728	Aconitate hydratase B	TCA cycle	*Alcaligenes*
Q1JW62	Aconitate hydratase B	TCA cycle	*Desulfuromonas*
I4N0T6	Aconitate hydratase B	TCA cycle	*Pseudomonas*
U7U739	Citrate synthase	TCA cycle	*Alcaligenes*
U7U7F2	Citrate synthase	TCA cycle	*Alcaligenes*
U7U7F8	Malate dehydrogenase	TCA cycle	*Alcaligenes*
Q74D54	Isocitrate dehydrogenase	TCA cycle	*Geobacter*
Q74EG8	Fumarate hydratase, class I	TCA cycle	*Geobacter*
I7FK92	Phosphoglycerate kinase	Glycolysis/gluconeogenesis	*Geobacter*
V9WRF6	Glyceraldehyde-3-phosphate dehydrogenase	Glycolysis/gluconeogenesis	*Pseudomonas*
A0A081GJT4	Glycogen debranching protein	Glycolysis/gluconeogenesis	*Cyanobium*
W7YDT5	Enolase	Glycolysis/gluconeogenesis	*Saccharicrinis*
N6YGD2	Phosphoenolpyruvate carboxykinase	Gluconeogenesis	*Thauera*
N6XEP4	Isocitrate lyase	Glyoxylate cycle	*Thauera*
	**Anaerobic Metabolism**		
Q74D51	2-oxoglutarate:ferredoxin oxidoreductase, alpha subunit (KorA)	Anaerobic TCA cycle	*Geobacter*
Q74D50	2-oxoglutarate:ferredoxin oxidoreductase, thiamin diphosphate-binding subunit (KorB)	Anaerobic TCA cycle	*Geobacter*
Q74D49	2-oxoglutarate:ferredoxin oxidoreductase, gamma subunit (KorC)	Anaerobic TCA cycle	*Geobacter*
Q74GZ6	Pyruvate-flavodoxin oxidoreductase (Por)	Anaerobic TCA cycle	*Geobacter*
E3PT29	Pyruvate-flavodoxin oxidoreductase (Por)	Anaerobic TCA cycle	*Clostridium*
B8J4R0	Sulfite reductase, dissimilatory-type alpha subunit	Anaerobic respiration	*Desulfovibrio*
B2YHF8	Dissimilatory sulphite reductase beta subunit (Fragment)	Anaerobic respiration	*Uncultured*

Q8EKJ1	Nitrate-inducible formate dehydrogenase molybdopterin-binding subunit FdnG	Anaerobic respiration	*Shewanella*
	**Acetate Metabolism**		
Q74FU6	NADPH-Fe(3+) oxidoreductase subunit alpha (SfrA)	Acetate metabolism.	*Geobacter*
Q74FU5	NADPH-Fe(3+) oxidoreductase subunit beta (SfrB)	Acetate metabolism.	*Geobacter*
Q74GS1	Succinyl:acetate coenzyme A transferase	Acetyl-CoA synthesis	*Geobacter*
	**Fatty Acid Metabolism**		
J0JLY0	Acetyl-CoA acetyltransferase	Fatty acid biosynthesis	*Alcaligenes*
Q74BM2	Acetyl-CoA carboxylase, biotin carboxylase component (Acc-ase)	Fatty acid biosynthesis	*Geobacter*
Q74CR7	3-oxoacyl-[acyl-carrier-protein] synthase 2 (EC 2.3.1.179)	Fatty acid biosynthesis	*Geobacter*
T0AZR6	Acyl carrier protein (ACP)	Fatty acid biosynthesis	*Thauera*
S9ZEL5	3-ketoacyl-ACP reductase (EC 1.1.1.36)	Fatty acid biosynthesis	*Thauera*
A0A022LGX0	Long-chain fatty acid--CoA ligase	Fatty acid β-oxidation	*Dietzia*
S9ZIA5	Acyl-CoA dehydrogenase	Fatty acid β-oxidation	*Thauera*
Q747G7	Biotin-dependent acyl-CoA carboxylase, carboxyltransferase subunit	Fatty acid β-oxidation	*Geobacter*
Q39UX8	Short-chain acyl-CoA dehydrogenase	Fatty acid β-oxidation	*Geobacter*
	**Membrane Proteins**		
U1XWB5	Membrane protein	Unknown	*Alcaligenes*
U7U8X8	Membrane protein	Unknown	*Alcaligenes*
X5HVW6	Membrane protein	Unknown	*Aeromonas*
H1RM45	Gram-negative type outer membrane porin protein	Transport	*Comamonas*
B9MDB0	Porin Gram-negative type	Transport	*Acidovorax*
X5HGU5	Porin	Transport	*Aeromonas*

A0A022LPU2	ABC transporter substrate-binding protein	Transport	*Dietzia*
A0A073ISB0	Branched-chain amino acid ABC transporter substrate-binding protein	Transport	*Synergistes*
L7X6F0	Membrane protein (Outer membrane protein A)	Receptor; transport	*Aeromonas*
Q74AK2	Sodium/solute symporter family protein	Transport	*Geobacter*
Q39Y99	Outer membrane channel, putative	Transport	*Geobacter*
Q74E95	Sodium/solute symporter family protein	Transport	*Geobacter*
K2N8B7	Basic membrane lipoprotein	Transport	*Nitratireductor*
K2PP47	Cationic amino acid ABC transporter periplasmic binding protein	Transport	*Nitratireductor*
W6XM61	ABC-type transporter, periplasmic subunit	Transport	*Burkholderia*
A0A075PCY9	Porin	Transport	*Pseudomonas*
Q74FD9	Lipoprotein cytochrome c	Electron transfer	*Geobacter*
Q74GH2	Cytochrome c,, and cytochrome b	Electron transfer	*Geobacter*
Q39RK5	ResB-like family cytochrome c	Electron transfer	*Geobacter*
Q74FJ5	ResB-like family cytochrome c biogenesis protein	Electron transfer	*Geobacter*
Q74GA2	NADH dehydrogenase I, G subunit	Electron transport chain	*Geobacter*
A0A067A3Q5	Outer membrane insertion C-terminal signal domain protein	Protein insertion	*Pseudomonas*
W8Q8M8	Outer membrane protein assembly factor BamA	Protein insertion	*Pseudomonas*
A0A077F872	Outer membrane protein H1	Antibiotic resistance	*Pseudomonas*
A0A081UU63	Maltoporin (Maltose-inducible porin)	Transport	*Aeromonas*
A0A085ETL9	Various polyols ABC transporter, periplasmic substrate-binding protein	Transport	*Devosia*
	**Stress Response or Interactions**		
Q74EI7	Phage tail sheath protein, putative	Phage	*Geobacter*
N6XZL4	Tail sheath protein	Phage	*Thauera*

Q74FS1	Cysteine synthase A	Contact-dependent inhibition	*Geobacter*
Q74E06	Superoxide dismutase	Oxidative stress	*Geobacter*
N6ZL72	Superoxide dismutase	Oxidative stress	*Thauera*
N6Y581	Phenylacetic acid degradation protein PaaD	Response to toxic aromatic	*Thauera*
U7UB80	Alkyl hydroperoxide reductase subunit C	Oxidative stress	*Alcaligenes*
Q74E46	Universal stress protein	Stress	*Geobacter*
	**Nitrogen Metabolism**		
E3PUH2	Glutamate dehydrogenase	Amino acid biosynthesis	*Clostridium*
G9PT06	Glutamate dehydrogenase	Amino acid biosynthesis	*Synergistes*
R5PD20	Glutamate dehydrogenase	Amino acid biosynthesis	*Odoribacter*
Q74DL1	Glutamate dehydrogenase	Amino acid biosynthesis	*Geobacter*
U7UGI7	Glutamate dehydrogenase	Amino acid biosynthesis	*Prevotella*
A0A022LGN0	Glutamate-binding protein	Amino acid biosynthesis	*Dietzia*
Q760A4	Nitrite reductase (Fragment)	Denitrification or nitrification	*uncultured*
U1YC31	Nitrous-oxide reductase	Denitrification	*Alcaligenes*
A1KA74	NosZ protein	Denitrification	*Azoarcus*
A7UMG8	Nitrous oxide reductase	Denitrification	*uncultured*
Q747H2	Rubredoxin:oxygen/nitric oxide oxidoreductase	Nitric oxide detoxification	*Geobacter*
Q74BM9	Nitrogen fixation protein NifU	Nitrogen fixation	*Geobacter*
E3PUA0	Hydroxylamine reductase/nitrate reductase (EC 1.7.99.1)	Nitrogen assimilation; nitric oxide production	*Clostridium*

Additional file 3

Table S6: Complete relative abundance values (mean %OTUs in a sample) for taxa in common between GhostKOALA annotation of proteins and OTUs from MiSeq sequencing of 16S rRNA gene amplicons from early and intermediate MFC anode biofilm samples. Relative abundances of OTUs are also shown for the solution and mature biofilm samples.

**Taxon**	**OTUs - solution**	**GhostKOALA-early**	**OTUs-early**	**GhostKOALA-intermediate**	**OTUs-intermediate**	**OTUs - mature**
Gammaproteobacteria - Others	84.31	74.49	83.17	27.90	25.41	1.02
Betaproteobacteria	3.36	8.83	1.40	19.95	11.62	1.48
Actinobacteria	0.12	2.63	1.55	8.38	9.95	2.91
Deltaproteobacteria	0.08	2.90	0.77	22.83	11.33	70.13
Bacteroidetes	0.54	2.66	5.17	4.84	6.34	0.73
Alphaproteobacteria	0.27	2.73	4.11	6.86	14.25	1.49
Epsilonproteobacteria	1.67	0.91	0.73	0.37	0.02	0.01
Gammaproteobacteria - Enterobacteria	6.57	1.83	0.26	1.73	0.43	0.08
Synergistetes	0.93	0.73	0.89	1.27	6.65	2.39
Firmicutes - Bacilli	0.02	0.63	0.11	0.41	0.44	0.14
Firmicutes - *Clostridia*	0.32	0.41	1.13	3.02	5.54	1.38
Cyanobacteria	0.00	0.26	0.00	0.74	0.00	0.00
Firmicutes - Others	0.04	0.21	0.13	0.67	0.51	0.28
Chrysiogenetes	0.00	0.16	0.00	0.00	0.00	0.00
Thermotogae	0.00	0.16	0.00	0.10	0.00	0.00
Fusobacteria	0.00	0.05	0.00	0.00	0.00	0.00
Spirochaetes	0.05	0.22	0.34	0.25	0.18	0.27
Chlamydiae	0.00	0.05	0.00	0.00	0.00	0.00
Planctomycetes	0.00	0.05	0.00	0.00	0.00	0.00
Chlorobi	0.00	0.05	0.00	0.05	0.00	0.00
Euryarchaeota	0.00	0.06	0.00	0.00	0.00	0.00
Caldiserica	0.00	0.06	0.00	0.00	0.00	0.00
Deferribacteres	0.00	0.00	0.00	0.17	0.00	0.00
Chloroflexi	0.00	0.00	0.00	0.10	0.00	0.00
Acidobacteria	0.00	0.00	0.00	0.12	0.00	8.74E-03
Gemmatimonadetes	0.00	0.00	0.00	0.12	0.01	7.03E-03
Verrucomicrobia	0.00	0.00	0.00	0.12	0.00	0.00
